# Visit and Between-Visit Interaction Frequency Before and After COVID-19 Telehealth Implementation

**DOI:** 10.1001/jamanetworkopen.2023.33944

**Published:** 2023-09-15

**Authors:** Sarah Nouri, Courtney R. Lyles, Elizabeth B. Sherwin, Magdalene Kuznia, Anna D. Rubinsky, Kathryn E. Kemper, Oanh K. Nguyen, Urmimala Sarkar, Dean Schillinger, Elaine C. Khoong

**Affiliations:** 1 Division of Palliative Medicine, Department of Medicine, University of California San Francisco; 2 Division of General Internal Medicine at Zuckerberg San Francisco General Hospital, Department of Medicine, University of California San Francisco; 3 UCSF Center for Vulnerable Populations, University of California San Francisco; 4 Department of Epidemiology and Biostatistics, University of California San Francisco; 5 University of California San Francisco School of Nursing; 6 Division of Hospital Medicine at Zuckerberg San Francisco General Hospital, Department of Medicine, University of California San Francisco

## Abstract

**Importance:**

Telehealth implementation associated with the COVID-19 public health emergency (PHE) affected patient–clinical team interactions in numerous ways. Yet, studies have narrowly examined billed patient-clinician visits rather than including visits with other team members (eg, pharmacists) or between-visit interactions.

**Objective:**

To evaluate rates of change over time in visits (in-person, telehealth) and between-visit interactions (telephone calls, patient portal messages) overall and by key patient characteristics.

**Design, Setting, and Participants:**

This retrospective cohort study included adults with diabetes receiving primary care at urban academic (University of California San Francisco [UCSF]) and safety-net (San Francisco Health Network [SFHN]) health care systems. Encounters from April 2019 to March 2021 were analyzed.

**Exposure:**

Telehealth implementation over 3 periods: pre-PHE (April 2019 to March 2020), strict shelter-in-place (April to June 2020), and hybrid-PHE (July 2020 to March 2021).

**Main Outcomes and Measures:**

The main outcomes were rates of change in monthly mean number of total encounters, visits with any health care team member, visits with billing clinicians, and between-visit interactions. Key patient-level characteristics were age, race and ethnicity, language, and neighborhood socioeconomic status (nSES).

**Results:**

Of 15 148 patients (4976 UCSF; 8975 SFHN) included, 2464 (16%) were 75 years or older, 7734 (51%) were female patients, 9823 (65%) self-identified as racially or ethnically minoritized, 6223 (41%) had a non-English language preference, and 4618 (31%) lived in the lowest nSES quintile. After accounting for changes to care delivery through an interrupted time-series analysis, total encounters increased in the hybrid-PHE period (UCSF: 2.3% per patient/mo; 95% CI, 1.6%-2.9% per patient/mo; SFHN: 1.8% per patient/mo, 95% CI, 1.3%-2.2% per patient/mo), associated primarily with growth in between-visit interactions (UCSF: 3.1% per patient/mo, 95% CI, 2.3%-3.8% per patient/mo; SFHN: 2.9% per patient/mo, 95% CI, 2.3%-3.4% per patient/mo). In contrast, rates of visits were stable during the hybrid-PHE period. Although there were fewer differences in visit use by key patient-level characteristics during the hybrid-PHE period, pre-PHE differences in between-visit interactions persisted during the hybrid-PHE period at SFHN. Asian and Chinese-speaking patients at SFHN had fewer monthly mean between-visit interactions compared with White patients (0.46 [95% CI, 0.42-0.50] vs 0.59 [95% CI, 0.53-0.66] between-visit interactions/patient/mo; *P* < .001) and English-speaking patients (0.52 [95% CI, 0.47-0.58] vs 0.61 [95% CI, 0.56-0.66] between-visit interactions/patient/mo; *P* = .03).

**Conclusions and Relevance:**

In this study, pre-PHE growth in overall patient-clinician encounters persisted after PHE-related telehealth implementation, driven in both periods by between-visit interactions. Differential utilization based on patient characteristics was observed, which may indicate disparities. The implications for health care team workload and patient outcomes are unknown, particularly regarding between-visit interactions. Therefore, to comprehensively understand care utilization for patients with chronic diseases, research should expand beyond billed visits.

## Key Points

**Question:**

How did use of visits and between-visit interactions change from April 2019 to March 2021, and did differences by age, race and ethnicity, language, or socioeconomic status that existed before the COVID-19 pandemic continue after COVID-19–related telehealth implementation?

**Findings:**

In this cohort study of 15 148 patients with diabetes, preexisting growth in total encounters persisted after COVID-19–related implementation, driven by growth in between-visit interactions. Pre–COVID-19 differences in visits by patient characteristics decreased, but differences in between-visit interactions persisted.

**Meaning:**

This study found that telehealth adoption affected utilization of both visits and between-visit interactions, with variation by patient characteristics; future research should explore the impact of growing between-visit interactions on equity, outcomes, and experience.

## Teaser

This cohort study evaluates rates of change over time in visits and between-visit interactions among US patients with diabetes before and after COVID-19 telehealth implementation.

## Introduction

The COVID-19 public health emergency (PHE) propelled the growth of telehealth, which we define as synchronous and asynchronous bidirectional remote patient-clinician communication.^1,2,3,4,5,6,7,8^ Flexible PHE-related policies initially meant to be temporary, such as reimbursement parity between in-person and video visits, continued for several years and have planned continuation in some situations even though the PHE has ended.^9,10,11,12^ The impact of increased telehealth adoption on outpatient patient-clinician interactions broadly among patients with chronic diseases remains unknown.

The gold standard of high-quality, personalized care for patients with chronic diseases is team-based care that takes place both during and between visits.^13,14,15,16^ *Visits* occur in-person or remotely by video or telephone and are typically scheduled in advance and billed. They occur most often between patients and physicians or advance practice practitioners (APPs) but also occur with nonbilling health care team members, such as nurses. *Between-visit interactions*, comprising unscheduled telephone calls and patient portal messages and generally used to address issues that arise between visits, can occur between patients and any health care team member and until very recently were rarely billed.^5,14,17,18,19,20^ While research examining changes in care utilization since the PHE is growing, existing studies have limited their scope to specific types of encounters (eg, billed visits), telehealth modalities (eg, patient portal messages), or clinician types (eg, physicians).^3,4,5,6,7,8,21,22,23,24,25,26,27,28,29,30,31,32^ Few studies have accounted for between-visit interactions and visits with nonbilling clinicians, likely due to reliance on payer data, which excludes most of these encounters.^3,21,24,31,33,34^ To gain a better understanding of changes in patterns of care for patients with chronic diseases, it is imperative to evaluate both visits and between-visit interactions.^18^

Furthermore, much of the literature does not distinguish the PHE shelter-in-place period from a subsequent “hybrid” period when telehealth and in-person visits were blended in routine care.^3,5,6,7,21,22,24,27^ Close attention to these distinct periods is needed because while telehealth visits substituted in-person care during the shelter-in-place period, both telehealth visits and in-person visits were available during the hybrid period. Moreover, given the wide variation across health systems in providing video or telephone-only telehealth visits, inclusion of safety-net settings, which often provide more telephone visits, in telehealth evaluations is particularly pressing.^5,6,21,22,35,36^ Safety-net systems serve a disproportionate share of disadvantaged patients who face barriers to digital health uptake. Moreover, they provide more nonreimbursed team-based care, which is poorly represented in studies relying on payer data.^37,38,39^

To our knowledge, no studies have assessed the impact of PHE-related telehealth implementation on the quantity of patient-health care team encounters overall. In this study, we evaluate rates of change in both visits and between-visit interactions among patients with diabetes with multiple health care team members in 2 health systems with different telehealth implementations. We examine these rates of change across 3 time periods (pre-PHE, shelter-in-place, and hybrid) to understand, after these abrupt changes to care delivery, the sustained rates of change in care utilization in the hybrid period. Diabetes, where team-based care has been shown to improve outcomes, is an ideal case study for chronic disease management managed with both visits and between-visit communication.^13,40,41,42^ We also compare patterns of differences by patient characteristics known to influence access to digital health care (age, preferred language, race and ethnicity, and neighborhood socioeconomic status [nSES])^43^ in the pre-PHE vs hybrid periods.

## Methods

### Study Setting and Data Source

Using electronic health record (EHR) data, we conducted a 2-year retrospective cohort study (April 1, 2019 to March 31, 2021) of patients with diabetes (eMethods in Supplement 1). All patients received primary care at 1 of 2 health care systems: University of California San Francisco (UCSF) or the San Francisco Health Network (SFHN). UCSF, an academic tertiary care center, has 4 primary care practices. SFHN, San Francisco County’s safety-net health system, has 14 primary care practices. The UCSF institutional review board approved this study. Informed consent was waived due to the low risk of the study and procedures involving only review of data collected for routine clinical care. This report follows the Strengthening the Reporting of Observational Studies in Epidemiology (STROBE) reporting guideline.

### Study Period

We evaluated 3 distinct time periods defined by health systems’ responses to the PHE: pre-PHE (April 2019 to March 2020 at UCSF; September 2019 to March 2020 at SFHN), strict PHE shelter-in-place (PHE-SIP; April to June 2020), and hybrid-PHE (July 2020 to March 2021). We used a different start date at SFHN due to adoption of a new EHR in August 2019. During PHE-SIP, health systems suspended nonemergency in-person services, and telehealth reimbursement parity began; thus, telehealth largely substituted in-person care. Hybrid-PHE began when health systems reintroduced nonemergency in-person services and telehealth reimbursement parity continued; therefore, both in-person and telehealth care were available.

### Study Sample

In each health system, we identified a cohort of patients (≥18 years old) with diabetes and empaneled in primary care as of April 1, 2019. We used EHR data to identify cohorts from problem lists, disease registries, laboratory results, and/or medications (eMethods in Supplement 1). To ensure patients were actively receiving care, we excluded those with no encounters from April 1, 2019, to March 31, 2021, with their primary care or endocrinology teams (hereafter jointly referred to as “health care team”). Encounters with the following team members were included: billing clinicians (physicians, APPs); nurses; social workers; pharmacists; dietitians; medical assistants; and embedded behavioral health clinicians.

### Variables

Healthcare utilization was measured at the patient level as mean number of encounters per patient per month, categorized into 4 outcomes (A to D) (eTable 1 in Supplement 1). Outcome A was total encounters, defined as any EHR-documented encounter (visit or between-visit interaction) between patients and any member of their health care team (ie, outcomes B and D combined). Visits consisted of scheduled in-person, video, or telephone appointments. Between-visit interactions consisted of all care that occurred between visits, documented in the EHR as unscheduled telephone calls or patient portal messages. Between-visit interactions can be initiated by patients or members of their health care team. A single between-visit interaction can include several back-and-forth calls or messages pertaining to the reason for the interaction (eg, one interaction could include a patient messaging to request a medication refill then the nurse forwarding the message with a comment to a doctor then the doctor replying to the patient that the medication has been refilled). Because they often involve multiple team members, we were unable to reliably identify between-visit interactions by clinician type. While patients with diabetes are advised to have a visit every 3 to 6 months (0.17-0.33 visits/patient/mo), there is no benchmark for the frequency of between-visit interactions. Outcome B was visits with any team member. Outcome C was visits with billing clinicians only (ie, a subset of outcome B). Outcome D was all between-visit interactions.

Key patient-level independent variables determined from the EHR were the following: (1) age (18-34, 35-49, 50-64, 65-74, ≥75 years); (2) race and ethnicity (Hispanic/Latinx, non-Hispanic Asian, non-Hispanic Black, non-Hispanic White, other non-Hispanic races, which included American Indian or Alaska Native, Native Hawaiian or Other Pacific Islander); (3) preferred language (English, Spanish, Chinese, other); and (4) nSES quintile based on geocoded residential addresses (eMethods in Supplement 1). We captured other patient data elements from the EHR: insurance; sex; Charlson Comorbidity Index, baseline diabetes control based on hemoglobin A_1c_ level, and baseline blood pressure (BP) control to adjust for differences in utilization based on medical need; and patient portal enrollment as a proxy for digital access (eMethods in the Supplement).

### Statistical Analysis

#### Descriptive Statistics

Each health system cohort was analyzed separately due to differences in patient population and primary telehealth modality. We used descriptive statistics to report all variables. For each of the 4 encounter-type outcomes, we report monthly means and the overall means within the pre-PHE and hybrid-PHE periods. For visits with any team member and between-visit interactions, we also report monthly means by modality.

#### Interrupted Time-Series Analyses

We used an interrupted time-series model with interruptions (intercept and slope changes) at the start of PHE-SIP and hybrid-PHE to model rates of change in outcomes A to D between study periods (pre-PHE vs PHE-SIP vs hybrid-PHE). While initial changes in care utilization at the start of the pandemic have been well documented, we chose an interrupted time-series model rather than an alternative analysis (eg, difference in differences) to focus our evaluation on sustained rates of change (slopes) in care utilization during the hybrid-PHE period. For each outcome A to D in the interrupted time-series analysis, we conducted Poisson regression models using generalized estimating equations with robust standard errors clustered on person (eMethods in Supplement 1). We used a multiple imputation by chained equations procedure (R version 4.2.1 [R Project for Statistical Computing]) to address missing values for baseline hemoglobin A_1c_ and BP measurements.^44^ Final models included all key patient-level independent variables and all covariates as well as slope changes at each interruption and statistically significant interaction terms between key independent variables and the PHE-SIP and hybrid-PHE intercept change variables (determined using Wald test with threshold of *P* < .05). We used model coefficients and 95% CIs to estimate the rate of change in encounters (slope) in the pre-PHE and hybrid-PHE periods and the magnitude of change (slope change) between the rates in these 2 periods.

#### Use in Pre-PHE vs PHE-Hybrid Periods by Patient-Level Characteristics

To examine differences by key patient-level independent variables in the pre-PHE and PHE-hybrid periods, we generated marginal means using ggeffects and plotted them using ggplot2.^45^ We conducted 2-sided pairwise comparisons of the estimated marginal means using the emmeans package.^46^ We used the Tukey method for pairwise comparisons (threshold of .05). Given the highly unusual nature of clinical care during the PHE-SIP period, we focused pairwise comparisons on the pre-PHE and PHE-hybrid periods.

## Results

### Included Participants

Final cohorts included 5268 patients at UCSF and 9880 patients at SFHN, for a total sample of 15 148 patients (Table). At UCSF, 1414 patients (27%) were 75 years or older; 2801 (53%) were female patients; 3312 (63%) self-identified as members of racially or ethnically minoritized groups; 967 (8%) had a non-English language preference; 2294 (44%) had Medicare insurance and 2270 (43%) had commercial insurance; 882 (17%) lived in the lowest nSES quintile; 1344 (26%) had at least 3 comorbid conditions; 972 (18%) had uncontrolled diabetes; and 1240 (24%) had uncontrolled hypertension. At SFHN, 1050 patients (11%) were 75 years or older; 4933 (50%) were female patients, 6511 (66%) self-identified as members of racially or ethnically minoritized groups; 5265 (53%) had a non-English language preference; 3062 (31%) had Medicare insurance and 3641 (37%) had Medicaid insurance; 3736 (38%) lived in the lowest nSES quintile; 1583 (16%) had at least 3 comorbid conditions; 1969 (20%) had uncontrolled diabetes; and 1718 (17%) had uncontrolled hypertension.

**Table.** Characteristics of Included Participants

Characteristic	Patients, No. (%)	
	UCSF (n = 5268)	SFHN (n = 9880)
Patient age, y		
18-34	169 (3.2)	272 (2.8)
35-49	639 (12.1)	1538 (15.6)
50-64	1607 (30.5)	4430 (44.8)
65-74	1439 (27.3)	2590 (26.2)
≥75	1414 (26.8)	1050 (10.6)
Sex		
Female	2801 (53.2)	4933 (49.9)
Male	2827 (46.8)	4947 (50.1)
Race and ethnicity^a^		
Hispanic	657 (12.5)	3280 (33.2)
Non-Hispanic Asian	1956 (37.1)	3369 (34.1)
Non-Hispanic Black	701 (13.3)	1558 (15.8)
Non-Hispanic White	1426 (27.1)	1159 (11.7)
Non-Hispanic American Indian or Alaska Native^b^	25 (0.5)	38 (0.4)
Non-Hispanic Native Hawaiian or Other Pacific Islander^b^	109 (2.1)	171 (1.7)
Other Non-Hispanic race and ethnicity	332 (6.3)	278 (2.8)
Language preference^a^		
English	4301 (81.6)	4624 (46.8)
Spanish	164 (3.1)	2606 (26.4)
Chinese	399 (7.6)	1731 (17.5)
Other	404 (7.7)	919 (9.3)
Insurance^c^		
Medicare	2294 (43.5)	3062 (31.0)
Medicaid	663 (12.6)	3641 (36.9)
Commercial	2270 (43.1)	182 (1.8)
Healthy Workers^d^	NA	1238 (12.5)
Uninsured^e^	2 (0.0)	1063 (10.8)
nSES quintile^c^		
1, Lowest	882 (16.7)	3736 (37.8)
2	959 (18.2)	2558 (25.9)
3	991 (18.8)	1465 (14.8)
4	1199 (22.8)	1284 (13.0)
5, Highest	976 (18.5)	593 (6.0)
Charlson Comorbidity Index		
0-2, No or mild comorbid illnesses	3924 (74.5)	8297 (84.0)
≥3, Moderate or severe comorbid illnesses	1344 (25.5)	1583 (16.0)
Baseline hemoglobin A_1c_, %^f^		
≤8, Controlled diabetes	3356 (63.7)	4232 (42.8)
>8, Uncontrolled diabetes	972 (18.5)	1969 (19.9)
Baseline blood pressure, mm Hg^f^		
≤120/80, No hypertension	936 (17.8)	2243 (22.7)
≤140/90, Controlled hypertension	2635 (50.0)	4793 (48.5)
>140/90, Uncontrolled hypertension	1240 (23.5)	1718 (17.4)
Enrolled in patient portal	3963 (75.2)	747 (7.6)

Abbreviations: NA, not applicable; nSES, neighborhood socioeconomic status; SFHN, San Francisco Health Network; UCSF, University of California San Francisco.

SI conversion factor: To convert hemoglobin A_1c_ to proportion of total hemoglobin, multiply by 0.01.

^a^ Participants with the following missing data were combined with “other” for analyses: race and ethnicity, 62 (1.2%) at UCSF and 27 (0.3%) at SFHN; language preference, 2 (<0.1%) at UCSF and 4 (<0.1%) at SFHN.

^b^ Due to small sample size, American Indian or Alaska Native and Native Hawaiian or Other Pacific Islander were grouped with other race and ethnicity in analyses.

^c^ Participants with missing data (301 [5.7%] at UCSF and 905 [9.2%] at SFHN) were excluded from interrupted time-series analyses. Individuals with the following data were excluded: insurance, 39 (0.7%) missing and 2 (<0.1%) self-pay at UCSF and 694 (7.0%) missing at SFHN; nSES, 261 (5.0%) at UCSF and 244 (2.5%) at SFHN; 2 (<0.1) missing sex at SFHN.

^d^ Healthy Workers is a city-provided insurance for home caregivers.

^e^ Uninsured at SFHN included individuals who had access to health care through local government access focused on specific populations (eg, undocumented individuals, incarcerated individuals).

^f^ Data presented is based on values where there were no missing data. As described in the Methods section, for analyses missing values were imputed for baseline A_1c_ (940 [17.8%] at UCSF; 3679 [37.2%] at SFHN) and baseline blood pressure (457 [8.7%] at UCSF; 1126 [11.4%] at SFHN).

### Care Utilization Over Time

#### Descriptive Patterns

**Figure 1.**
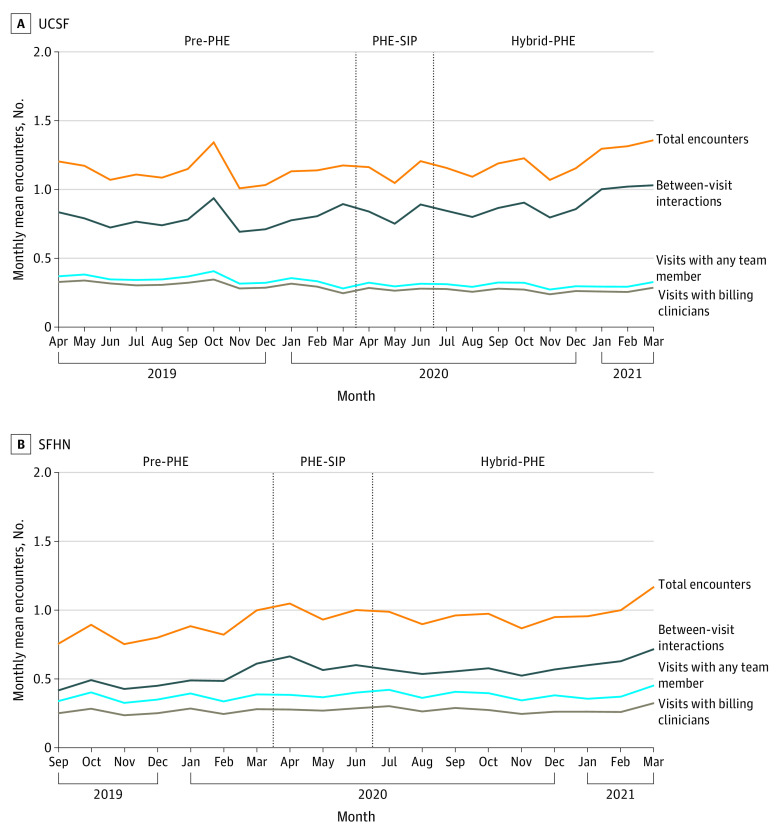
Mean Number of Patient-Clinician Encounters per Month Among 5268 Patients Receiving Care at University of California San Francisco (UCSF) and 9880 Patients Receiving Care at San Francisco Health Network (SFHN) From April 2019 to March 2021 Visits with any team member include in-person, video, or phone visits with any member of the care team. Visits with billing clinicians include any in-person, video, or phone visit with billing clinicians (physicians, advance practice practitioners). Between-visit interactions include any unscheduled phone call or patient portal message with any member of the care team. Total encounters equals visits with any team member and between-visit interactions. Pre-PHE indicates pre–COVID-19 public health emergency, April 1, 2019, to March 31, 2020, at UCSF; September 1, 2019, to March 31, 2020, at SFHN; PHE-SIP, public health emergency shelter in place, April 1 to June 30, 2020; and hybrid-PHE, hybrid public health emergency, July 1, 2020, to March 31, 2021.

Figure 1 shows monthly means of the 4 outcomes at UCSF and SFHN: total encounters (outcome A), visits with any team member (outcome B), visits with billing clinicians (outcome C), and between-visit interactions (outcome D). The overall mean (SD) number of total encounters per patient per month was higher in the hybrid-PHE period than in the pre-PHE period at both sites (UCSF, pre-PHE vs hybrid-PHE: 1.13 [0.09] per mo vs 1.21 [0.10] per mo; SFHN, pre-PHE vs hybrid-PHE: 0.84 [0.09] per mo vs 1.00 [0.08] per mo).

**Figure 2.**
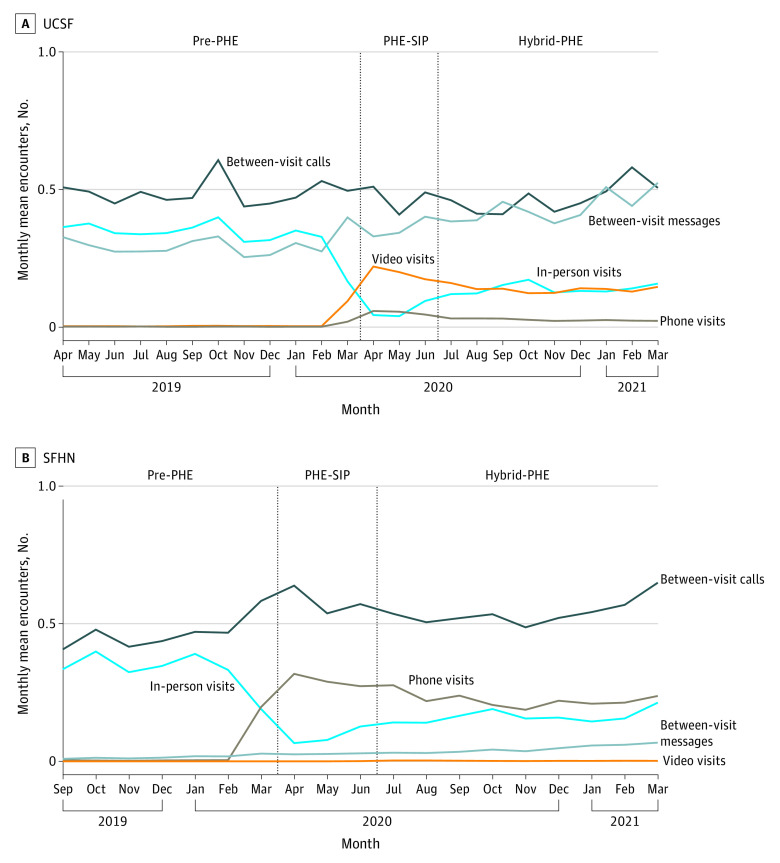
Mean Number of Patient-Clinician Encounters by Visit and Between-Visit Interaction Modality per Month Among 5268 Patients Receiving Care at University of California San Francisco (UCSF) and 9880 Patients Receiving Care at San Francisco Health Network (SFHN) From April 2019 to March 2021 In-person, video, and phone visits are scheduled visits; between-visit calls and between-visit messages are unscheduled between-visit interactions. Pre-PHE indicates pre–COVID-19 public health emergency, April 1, 2019, to March 31, 2020, at UCSF and September 1, 2019, to March 31, 2020, at SFHN; PHE-SIP, public health emergency shelter in place, April 1 to June 30, 2020; and hybrid-PHE, hybrid public health emergency, July 1, 2020, to March 31, 2021.

Mean (SD) visits with any team member and with billing clinicians decreased at UCSF in the pre-PHE vs hybrid-PHE periods (any team member: 0.35 [0.03] per mo vs 0.30 [0.02] per mo; billing clinicians: 0.31 [0.03] per mo vs 0.26 [0.01] per mo). Mean (SD) visits with any team member increased slightly at SFHN between the pre-PHE and hybrid-PHE periods (0.36 [0.03] per mo to 0.39 [0.03] per mo), while visits with billing clinicians remained stable. At the start of PHE-SIP, in-person visits were replaced mostly by video visits at UCSF and telephone visits at SFHN, although by the end of hybrid-PHE, in-person visits occurred at approximately the same rate as video visits at UCSF and as phone visits at SFHN (Figure 2).

In both health systems, the increase in total encounters over time was driven largely by an increase in between-visit interactions, which comprised more than half of all encounters in both systems in most periods (79%-90% at UCSF, 48%-61% at SFHN). Mean (SD) between-visit interactions per patient per month increased 14% at UCSF (from 0.79 [0.07] per mo pre-PHE to 0.90 [0.09] per mo hybrid-PHE) and 23% at SFHN (from 0.48 [0.06] per mo pre-PHE to 0.59 [0.06] per mo hybrid-PHE). At UCSF (Figure 2), this growth was due to an increase in mean (SD) patient portal messages (from 0.30 [0.04] per mo pre-PHE to 0.43 [0.05] per mo hybrid-PHE). SFHN had an increase in both mean (SD) unscheduled telephone calls (from 0.47 [0.06] per mo pre-PHE to 0.54 [0.05] per mo hybrid-PHE) and patient portal messages (from 0.02 [0.006] per mo pre-PHE to 0.05 [0.01] per mo hybrid-PHE) (Figure 2).

#### Adjusted Analyses

At UCSF, total encounters were relatively stable in the pre-PHE period (−0.2% per patient/mo; 95% CI, −0.6% to 0.3% per patient/mo) but increased during the hybrid-PHE period (2.3% per patient/mo; 95% CI, 1.6% to 2.9% per patient/mo; estimated 27 encounters/1000 patients/mo) (eFigure 1 and eTables 2-4 in Supplement 1). This represents a 2.4% (95% CI, 1.6% to 3.2%) increase in the slope from the pre-PHE period to the hybrid-PHE period. The growth in total encounters during the hybrid-PHE period was driven by between-visit interactions, which increased 2.6% (95% CI, 1.7% to 3.6%) from the pre-PHE to the hybrid-PHE periods (pre-PHE: 0.5% per patient/mo; 95% CI, −0.1% to 1.0%; estimated 4 encounters/1000 patients/mo; hybrid-PHE: 3.1% per patient/mo; 95% CI, 2.3% to 3.8%; estimated 28 encounters/1000 patients/mo). Rates of visits with any team member and billing clinicians were decreasing during the pre-PHE period (any team member: −1.5% per patient/mo; 95% CI, −1.9% to −1.1% per patient/mo; billing clinician: −1.6% per patient/mo; 95% CI, −2.0% to −1.2% per patient/mo; estimated 6 visits/1000 patients/mo for both) but stabilized during the hybrid-PHE period (any team member: −0.1% per patient/mo; 95% CI, −0.8% to 0.6% per patient/mo; billing clinician: −0.2% per patient/mo; 95% CI, −0.9% to 0.7% per patient/mo).

At SFHN, the rate of total encounters was increasing in both the pre-PHE and hybrid-PHE periods (pre-PHE: 3.1% per patient/mo; 95% CI, 2.5% to 3.8% per patient/mo; estimated 25 encounters/1000 patients/mo; hybrid-PHE: 1.8% per patient/mo; 95% CI, 1.3% to 2.2% per patient/mo; estimated 18 encounters/1000 patients/mo) (eFigure 1 and eTable 4 in Supplement 1) although there was a 1.3% (95% CI, 0.5% to 2.1%) decrease in the slope from the pre-PHE period to the hybrid-PHE period. Similarly, the rate of between-visit interactions was increasing in both periods (pre-PHE: 4.9% per patient/mo; 95% CI, 4.1% to 5.7% per patient/mo; estimated 21 interactions/1000 patients/mo; hybrid-PHE 2.9% per patient/mo; 95% CI, 2.3% to 3.4% per patient/mo; estimated 17 interactions/1000 patients/mo), although there was a 1.9% (95% CI, 1.0% to 2.8%) decrease in the slope from the pre-PHE period to the hybrid-PHE period. Rates of visits with any team member and billing clinicians were increasing during the pre-PHE period (any team member: 0.9% per patient/mo; 95% CI, 0.2% to 1.6% per patient/mo; billing clinician: 1.0% per patient/mo; 95% CI, 0.3% to 1.7% per patient/mo; estimated 3 visits/1000 patients/mo for both) and were stable during the hybrid-PHE period (any team member: 0.2% per patient/mo; 95% CI, −0.4% to 0.7% per patient/mo; billing clinician: 0.1%; 95% CI, −0.5% to 0.6% per patient/mo).

### Differential Utilization Across Time by Key Demographic Characteristics

Differences by key patient-level characteristics (age, race and ethnicity, language, nSES) in outcomes A to D within each period based on marginal means are presented in eTable 5 in Supplement 1. Figures of interrupted time-series analyses stratified by age, race and ethnicity, and language are presented in eFigures 2 to 7 in Supplement 1.

#### Age

There were no significant differences by age at UCSF. At SFHN, younger individuals had fewer total encounters, visits with billing clinicians, and between-visit interactions during the pre-PHE period compared with older individuals (eFigure 8 in Supplement 1); these differences were no longer significant during the hybrid-PHE period. During the hybrid-PHE period, patients aged 50 to 64 years had more visits with any team member compared with those aged 75 years or older.

#### Race and Ethnicity

**Figure 3.**
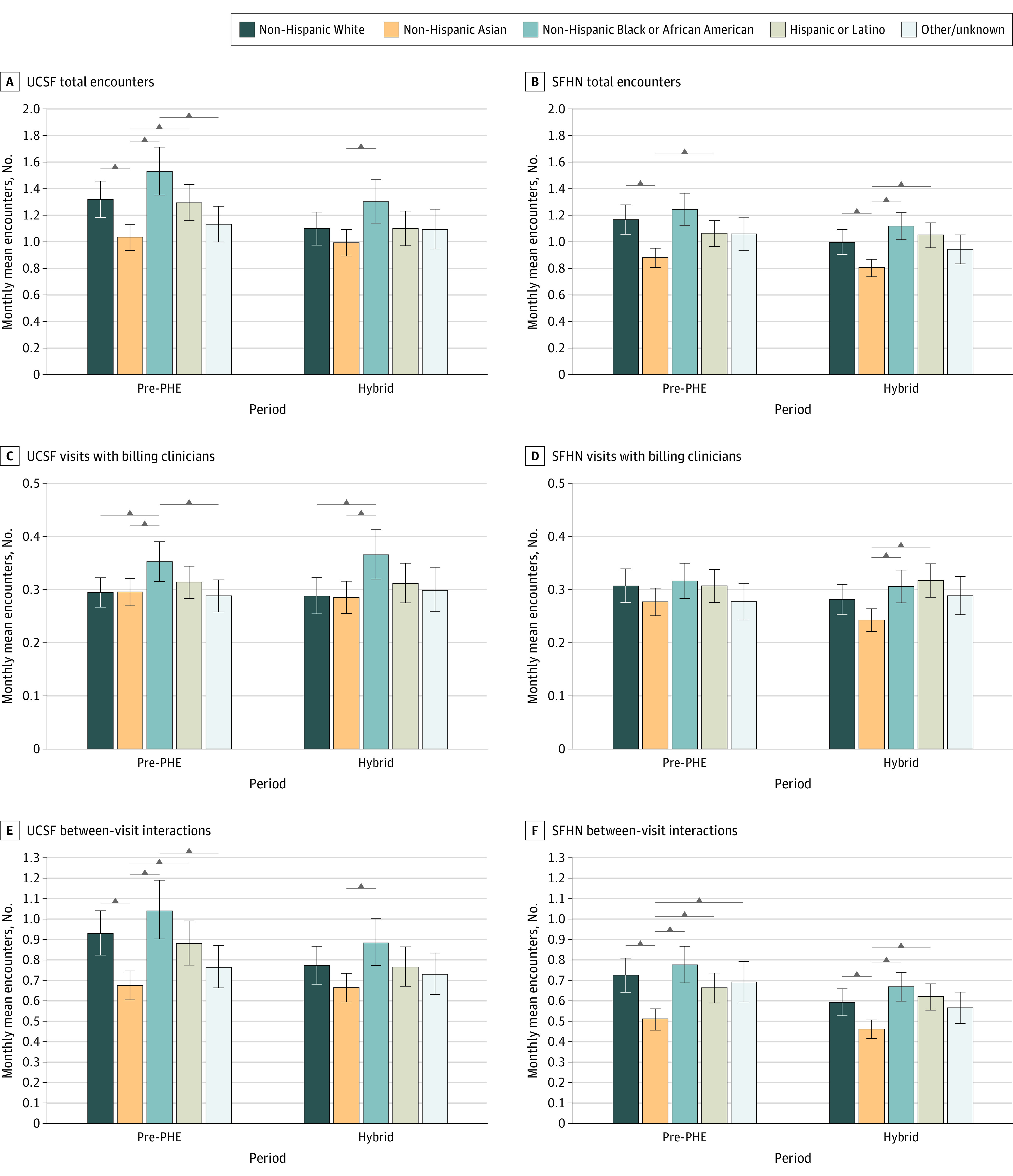
Adjusted Encounter Means by Race and Ethnicity in the Pre–Public Health Emergency (PHE) and Hybrid-PHE Periods Among 4967 Patients Receiving Care at University of California San Francisco (UCSF) and 8975 Patients Receiving Care at San Francisco Health Network (SFHN) Pre-PHE indicates April 1, 2019, to March 31, 2020, at UCSF and September 1, 2019, to March 31, 2020, at SFHN; hybrid, July 1, 2020 to March 31, 2021. Horizontal bars indicate significant difference at *P* < .05 (all comparisons tested). Error bars indicate the 95% CI for the adjusted mean.

Patients identifying as Asian had fewer total encounters and between-visit interactions compared with those identifying as non-Hispanic White, Black, or Hispanic/Latinx during the pre-PHE period at both UCSF and SFHN (Figure 3). These differences persisted in the hybrid-PHE period at SFHN; specifically Asian patients had fewer monthly mean between-visit interactions compared with White patients (0.46 [95% CI, 0.42-0.50] vs 0.59 [95% CI, 0.53-0.66] between-visit interactions/patient/mo; *P* < .001). However, only the difference between Asian and Black patients remained significant in the hybrid-PHE period at UCSF. At UCSF, patients identifying as Black had more visits (with billing clinicians and any team member) compared with non-Hispanic White and Asian in all periods (eFigure 9 in Supplement 1).

#### Language Preference

**Figure 4.**
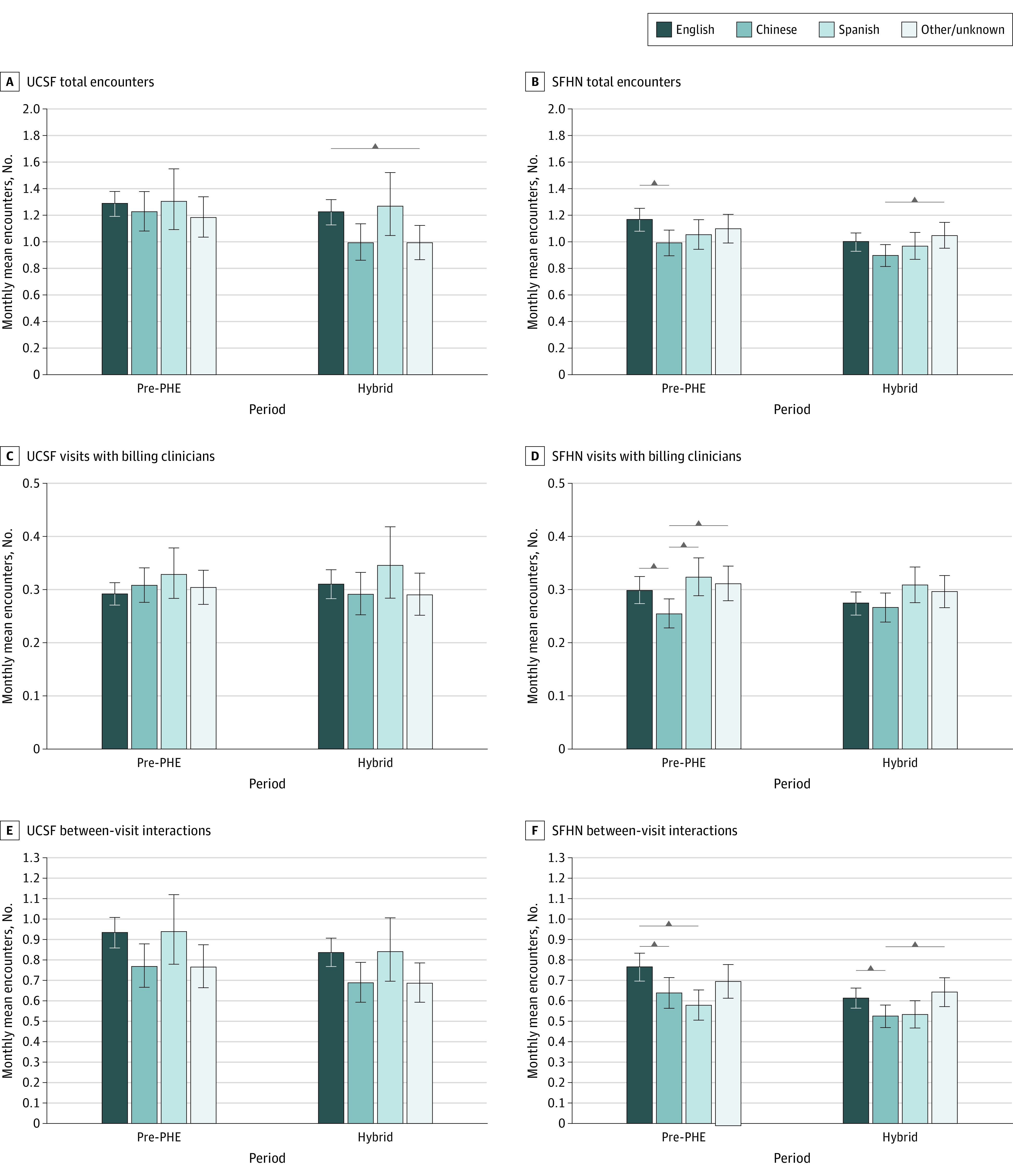
Adjusted Encounter Means by Language Preference in the Pre–Public Health Emergency (PHE) and Hybrid-PHE Periods Among 4967 Patients Receiving Care at University of California San Francisco (UCSF) and 8975 Patients Receiving Care at San Francisco Health Network Pre-PHE indicates April 1, 2019, to March 31, 2020, at UCSF and September 1, 2019, to March 31, 31, 2020; hybrid-PHE, July 1, 2020 to March 31, 2021. Horizontal bars indicate significant difference at *P* < .05 (all comparisons tested). Error bars indicate the 95% CI for the adjusted mean.

At UCSF, there were no differences by language in any outcome in the pre-PHE period (Figure 4 and eFigure 10 in Supplement 1). However, in the hybrid-PHE period, patients with a preferred language other than English, Spanish, or Chinese had fewer total encounters compared with English speakers. At SFHN in the pre-PHE period, Chinese speakers had fewer total encounters and between-visit interactions compared with English speakers and fewer visits with billing clinicians compared with all other groups. This difference persisted for between-visit interactions in the hybrid-PHE period (0.52 [95% CI, 0.47-0.58] vs 0.61 [95% CI, 0.56-0.66] between-visit interactions/patient/mo; *P* = .03), but not for visits with billing clinicians. Spanish speakers had fewer between-visit interactions in the pre-PHE period compared with English speakers, but this difference was no longer significant in hybrid-PHE.

#### nSES

There were no differences in outcomes A to D by nSES at either site. Results appear in eTables 2 and 3 in Supplement 1.

## Discussion

Despite different telehealth implementation strategies and patient populations in the academic (UCSF) and safety-net (SFHN) settings, total encounters continued to increase significantly during the hybrid period at both health systems, driven by between-visit interactions. These between-visit interactions—primarily patient messages at UCSF and a combination of telephone calls and patient messages at SFHN—are crucial to advancing health, especially when team-based care is the standard of care, but they are understudied.^18,28^ Within our investigation of differences in utilization by demographic characteristics, we found fewer differences in visit frequency by age and language preference during the hybrid-PHE period compared with the pre-PHE period at SFHN. In contrast, among between-visit interactions, differences persisted by language preference, decreased by age at SFHN, and decreased by race and ethnicity at UCSF.

Prior literature has shown recent growth in patient portal messages.^25,26^ We found that unscheduled telephone calls have also been growing, particularly in the safety-net system.^26,47^ Work is needed to understand the cause and impact of the growth in between-visit interactions, which address care concerns between scheduled visits.^18^ This growth may be because synchronous telehealth visits are inadequate and require more between-visit care, patients do not have timely access to clinician visits, or visits are inconvenient, suggesting between-visit interactions may be an important access point for patients who would otherwise forego care. Notably, prior literature has demonstrated that growth in patient portal messages is associated with higher clinician workload.^48,49,50^ To alleviate clinician burnout associated with these increased tasks, some payers have begun reimbursing some patient portal messages.^51^ However, similar attention has not been paid to the growing number of telephone-based between-visit interactions, nor has attention been paid to the impact of this growing between-visit workload on clinical teams more broadly. The uptake by clinicians of billing for patient portal messages remains low, similar to the past introduction of other billing codes.^51,52^ Billing for patient portal messages may inhibit their use due to copays, further limiting access for patients.^53^ Thus, while the best approach to cope with the growing number of between-visit interactions remains unclear, our findings indicate that their impact on clinician well-being and health outcomes merit examination.

Of particular importance to equity is our finding that utilization patterns by sociodemographic characteristics differed during the pre-PHE period compared with the hybrid-PHE period and by encounter type. Differences in visits by race and ethnicity and preferred language that existed during the pre-PHE period disappeared during the hybrid-PHE period, except for Black patients, who continued to have more visits than other patients. Prior studies have expressed concerns about equity in telehealth access.^23,54^ We found that when a mix of in-person, video, and telephone options were available during the hybrid-PHE period, there were fewer differences in utilization by race and ethnicity and preferred language compared with when visits were primarily conducted in person, suggesting that telehealth options may reduce disparities. Adults younger than 35 years at SFHN had fewer between-visit interactions compared with older patients during the pre-PHE period, but this difference was no longer present during the hybrid-PHE period. This may represent greater engagement of younger patients via telehealth and/or barriers to telehealth utilization among older adults due to lower digital literacy. While both Chinese speakers and Spanish speakers had a lower frequency of between-visit interactions at SFHN compared with English speakers during the pre-PHE period, this difference persisted over time for Chinese speakers but disappeared for Spanish speakers during the hybrid-PHE period, possibly because language access for Chinese speakers remains limited on patient portals or when patients call health care systems.^55^

### Limitations

Our study is limited by only including 2 urban health systems and data through March 2021, which may not be generalizable to later periods during the pandemic when concerns over virus exposure decreased. In identifying our cohorts, the accuracy of EHR problem lists or medication lists is unknown; we tried to mitigate this through medical record reviews for data validation. We could not account for unmeasured confounders, such as losing access to health care due to PHE-related job loss. New EHR adoption with a new patient portal at SFHN in late 2019 may have impacted our results, though less than 5% of patients had active patient portal accounts in the prior EHR. We did not have access to data beyond our 2 health systems, so we could not account for patients having external encounters; we restricted our cohorts to patients actively receiving care at UCSF or SFHN to mitigate this.

## Conclusions

In these 2 urban health care systems, patients with diabetes were increasingly engaging with their health care teams through between-visit patient messages or telephone calls. In contrast, visits, both with billing clinicians and with any team member, were stable during the hybrid-PHE period. Patterns of utilization by key sociodemographic characteristics differed in the hybrid-PHE period compared with the pre-PHE period, with nuanced implications for health equity. More research is needed to evaluate the drivers and impact of between-visit interactions, particularly on clinical outcomes and clinician well-being, including among nonphysician and APP members of the health care team. Our findings encourage broadening current conversations regarding the impact of telehealth on care utilization to consider how a range of encounter types can be optimized to ensure equitable access to team-based care for chronic disease management.

## Notes

Supplement 1. **eMethods.** Methodological Supplement **eTable 1.** Encounter Types **eTable 2.** Interrupted Time-Series Model Coefficients at UCSF **eTable 3.** Interrupted Time-Series Model Coefficients at SFHN **eTable 4.** Slope and Slope Change Incident Rate Ratios **eTable 5.** Estimated Marginal Means **eFigure 1.** Interrupted Time-Series Analysis of Patient-Clinician Encounters per Month at UCSF (n = 4967) and SFHN (n = 8975) **eFigure 2.** Interrupted Time-Series Analysis of Patient-Clinician Encounters per Month at UCSF (n = 4967) Stratified by Patient Age **eFigure 3.** Interrupted Time-Series Analysis of Patient-Clinician Encounters per Month at SFHN (n = 8975) Stratified by Patient Age **eFigure 4****.** Interrupted Time-Series Analysis of Patient-Clinician Encounters per Month at UCSF (n = 4967) Stratified by Patient Race and Ethnicity **eFigure 5****.** Interrupted Time-Series Analysis of Patient-Clinician Encounters per Month at SFHN (n = 8975) Stratified by Patient Race and Ethnicity **eFigure 6.** Interrupted Time-Series Analysis of Patient-Clinician Encounters per Month at UCSF (n = 4967) Stratified by Patient Language **eFigure 7****.** Interrupted Time-Series Analysis of Patient-Clinician Encounters per Month at SFHN (n = 8975) Stratified by Patient Language **eFigure 8.** Adjusted Encounter Means by Age in Pre-PHE and Hybrid-PHE Periods at UCSF (n = 4967) and SFHN (n = 8975) **eFigure 9.** Adjusted Encounter Means by Race and Ethnicity for Visits With Any Team Member in Pre-PHE and Hybrid-PHE Periods at UCSF (n = 4967) and SFHN (n = 8975) **eFigure 10.** Adjusted Encounter Means by Language for Visits With Any Team Member in Pre-PHE and Hybrid-PHE Periods at UCSF (n = 4967) and SFHN (n = 8975)

Supplement 2.
Data Sharing Statement
