# Supplementary material for: Visit and Between-Visit Interaction Frequency Before and After COVID-19 Telehealth Implementation
Source: JAMA Netw Open. 2023 Sep 15;6(9):e2333944. doi: 10.1001/jamanetworkopen.2023.33944 (PMC10504619; doi:10.1001/jamanetworkopen.2023.33944)
Supplement: Supplement 1. — eMethods. Methodological Supplement eTable 1. Encounter Types eTable 2. Interrupted Time-Series Model Coefficients at UCSF eTable 3. Interrupted Time-Series Model Coefficients at SFHN eTable 4. Slope and Slope Change Incident Rate Ratios eTable 5. Estimated Marginal Means eFigure 1. Interrupted Time-Series Analysis of Patient-Clinician Encounters per Month at UCSF (n = 4967) and SFHN (n = 8975) eFigure 2. Interrupted Time-Series Analysis of Patient-Clinician Encounters per Month at UCSF (n = 4967) Stratified by Patient Age eFigure 3. Interrupted Time-Series Analysis of Patient-Clinician Encounters per Month at SFHN (n = 8975) Stratified by Patient Age eFigure 4. Interrupted Time-Series Analysis of Patient-Clinician Encounters per Month at UCSF (n = 4967) Stratified by Patient Race and Ethnicity eFigure 5. Interrupted Time-Series Analysis of Patient-Clinician Encounters per Month at SFHN (n = 8975) Stratified by Patient Race and Ethnicity eFigure 6. Interrupted Time-Series Analysis of Patient-Clinician Encounters per Month at UCSF (n = 4967) Stratified by Patient Language eFigure 7. Interrupted Time-Series Analysis of Patient-Clinician Encounters per Month at SFHN (n = 8975) Stratified by Patient Language eFigure 8. Adjusted Encounter Means by Age in Pre-PHE and Hybrid-PHE Periods at UCSF (n = 4967) and SFHN (n = 8975) eFigure 9. Adjusted Encounter Means by Race and Ethnicity for Visits With Any Team Member in Pre-PHE and Hybrid-PHE Periods at UCSF (n = 4967) and SFHN (n = 8975) eFigure 10. Adjusted Encounter Means by Language for Visits With Any Team Member in Pre-PHE and Hybrid-PHE Periods at UCSF (n = 4967) and SFHN (n = 8975) [file jamanetwopen-e2333944-s001.pdf]

Supplemental Online Content

Nouri S, Lyles CR, Sherwin EB, et al. Visit and between-visit interaction frequency before and after COVID-19 telehealth implementation. *JAMA Netw Open*. 2023;6(9):e2333944.
doi:10.1001/jamanetworkopen.2023.33944

eMethods. Methodological Supplement
eTable 1. Encounter Types
eTable 2. Interrupted Time-Series Model Coefficients at UCSF
eTable 3. Interrupted Time-Series Model Coefficients at SFHN
eTable 4. Slope and Slope Change Incident Rate Ratios
eTable 5. Estimated Marginal Means
eFigure 1. Interrupted Time-Series Analysis of Patient-Clinician Encounters per Month at UCSF (n = 4967) and SFHN (n = 8975)
eFigure 2. Interrupted Time-Series Analysis of Patient-Clinician Encounters per Month at UCSF (n = 4967) Stratified by Patient Age
eFigure 3. Interrupted Time-Series Analysis of Patient-Clinician Encounters per Month at SFHN (n = 8975) Stratified by Patient Age
eFigure 4. Interrupted Time-Series Analysis of Patient-Clinician Encounters per Month at UCSF (n = 4967) Stratified by Patient Race and Ethnicity
eFigure 5. Interrupted Time-Series Analysis of Patient-Clinician Encounters per Month at SFHN (n = 8975) Stratified by Patient Race and Ethnicity
eFigure 6. Interrupted Time-Series Analysis of Patient-Clinician Encounters per Month at UCSF (n = 4967) Stratified by Patient Language
eFigure 7. Interrupted Time-Series Analysis of Patient-Clinician Encounters per Month at SFHN (n = 8975) Stratified by Patient Language
eFigure 8. Adjusted Encounter Means by Age in Pre-PHE and Hybrid-PHE Periods at UCSF (n = 4967) and SFHN (n = 8975)
eFigure 9. Adjusted Encounter Means by Race and Ethnicity for Visits With Any Team Member in Pre-PHE and Hybrid-PHE Periods at UCSF (n = 4967) and SFHN (n = 8975)
eFigure 10. Adjusted Encounter Means by Language for Visits With Any Team Member in Pre-PHE and Hybrid-PHE Periods at UCSF (n = 4967) and SFHN (n = 8975)

This supplemental material has been provided by the authors to give readers additional information about their work.

© 2023 Nouri S et al. *JAMA Network Open.*

eMethods. Methodological Supplement

Details on cohort definition

Cohort Inclusion Criteria

1. Active primary care panel patient, defined as:
    a. A primary care clinician listed as primary PCP in Apex, AND
    b. Any in-person, telephone, telehealth, or MyChart encounter with a primary care department since 03/01/2016
2. Patients 18+ years old as of 03/01/2019
3. With diabetes diagnosis based on any of the following criteria:
    a. Having an active diabetes mellitus health maintenance modifier on 3/1/2019
    b. ICD-9/ICD-10 codes on active problem list as of 3/1/2019
        i. ICD-9: '250', '357.2', '362.01', '362.02', '362.03', '362.04', '362.05', '362.06', '362.07', '366.41', '648.00', '648.01', '648.02', '648.03', '648.04'
        ii. ICD-10: 'E10', 'E11', 'E13'
    c. Most-recent A1c test result within 3-year span of 3/1/2016 - 3/1/2019:
        i. HbA1c > 6.5%, or
        ii. HgA1c < 6.5% with an active diabetes medication on medication list:
            (a) Any non-metformin medication, or
            (b) A combination medication including metformin
            Medication list: 'acarbose', 'actoplus', 'actos', 'amaryl', 'apidra', 'avandamet', 'avandaryl', 'avandia', 'bydureon', 'byetta', 'chlorpropamide', 'dapagliflozin', 'diabeta', 'duetact', ' dulaglutide ', 'farxiga', 'glimepiride', 'glipizide', 'glucotrol', 'glucovance', 'glyburide', 'glynase', 'glyset', 'humalog', 'humulin', 'invokamet', 'invokana', 'janumet', 'januvia', 'jardiance', 'jentadueto', 'juvisync', 'kazano', 'kombiglyze', 'lantus', 'levemir', 'liraglutide', 'lispro', 'metaglip', 'metformin' combo, 'nateglinide', 'nesina', 'novolin', 'novolog', 'onglyza', 'oseni', 'pioglitazone', 'prandimet', 'prandin%', 'precose', 'relion', 'repaglinide', 'starlix', 'symlin', 'tanzeum', 'tolazamide', 'tolbutamide', 'tradjenta', 'trulicity', 'victoza'

Cohort Exclusion Criteria

1. Diabetes diagnosis specifically for:
    a. Gestational diabetes
    b. Pre-diabetes
    c. Steroid-induced diabetes
2. Health maintenance modifier stating: "Exclude from Diabetes Registry: Does not have Diabetes"

Additional Definitions

1. Charlson comorbidity score restricted to diagnoses entered between 1-year period from 3/1/2019 – 3/1/2020.

Details about covariates

Sociodemographic characteristics

The following were determined as of 4/1/2019: (a) age (18-34, 35-49, 50-64, 65-74, ≥75); (b) sex (male, female); (c) race/ethnicity (Asian, Black, Latinx, non-Hispanic White, Other non-Hispanic races, which included American Indian/Alaska Native, Native Hawaiian or other Pacific Islander); (d) preferred language (English, Spanish, Chinese, other); (e) insurance type/status (Medicare [includes dual eligible Medicare and Medicaid]; Medicaid; commercial; Healthy Workers [a city-provided insurance for home caregivers]; uninsured, self-pay, or local government access programs); and (f) neighborhood socioeconomic status (nSES) quintile based on geocoded addresses. nSES is a composite indicator that incorporates the most recent 5-year averages from the 2013 to 2017 American Community Survey data on income, education, poverty, employment, occupation, housing and rent values, applied to 2010 US Census tract boundaries.[1] We determined nSES by linking patient addresses from the EHR to census tract. We created quintiles of nSES based on the distribution of nSES across US Census tracts of SF Bay Area counties, with the first quintile (Q1) reflecting lowest nSES and the fifth quintile (Q5) reflecting highest nSES US Census tracts.

[1] Gomez SL, Glaser SL, McClure LA, Shema SJ, Kealey M, Keegan TH, Satariano WA. The California Neighborhoods Data System: a new resource for examining the impact of neighborhood characteristics on cancer incidence and outcomes in populations. Cancer Causes & Control. 2011 Apr;22(4):631-47.

© 2023 Nouri S et al. *JAMA Network Open.*

Health-related trait

(a) Charlson co-morbidity index (modified to exclude diabetes by convention) as of 4/1/2019; (b) baseline diabetes control based on most recent hemoglobin A1c from 4/1/2019-2020, categorized as controlled (A1c ≤8) vs uncontrolled (A1c >8); (c) baseline blood pressure (BP) control based on the mean of the 3 most recent outpatient BP measurements from 4/1/2019-2020, categorized as ≤120/80 (no hypertension), >120/80 and ≤140/90 (controlled hypertension), >140/90 (uncontrolled hypertension).

Digital access

Digital access was defined as enrollment in patient portal as of 4/1/2020.

Details about analytic approach

For variables with >10% missingness (baseline A1c [n=940 (18%) at UCSF; n=3679 (37%) at SFHN] and baseline BP [n=457 (9%) at UCSF; n=1126 (11%) at SFHN]) we conducted logistic regressions to determine if covariates were associated with missingness of these variables. We found that several covariates were associated with missing baseline A1c and/or baseline BP and thus the data were not missing completely at random (MCAR) and a complete case analysis would not be unbiased. We used a multiple imputation by chained equations (MICE) procedure to address missing values for baseline A1c and baseline blood pressure. Imputation was performed using the MICE package in R 4.2.1 (R Core Team, Vienna, Austria).[2] Sensitivity analyses with complete case were conducted for all models. Interaction term coefficients from complete case analyses were consistent with results from multiple imputation (same direction of effect) although there were several differences in significance.

For each outcome of number of encounters per person per month we conducted Poisson regression analyses using generalized estimating equations with robust standard errors clustered on person. For each interruption we generated an intercept change variable equal to zero before the interruption and one after the interruption, and a slope change variable equal to zero before the interruption and number of months after the interruption after. The first interruption, PHE-SIP, started on 4/1/2020 and the second interruption, hybrid-PHE, started on 7/1/2020. Each intercept change and slope change in eTable 2 and eTable 3 represent the change from the previous period. Model coefficients (eTable 2 and eTable 3) were used to calculate incident rate ratios and 95% confidence intervals for slopes during each period as well as slope changes from Pre-PHE to Hybrid-PHE (eTable 4) using the delta method in the car package.[3]

We evaluated interactions between each key predictor variable and the PHE-SIP and hybrid-PHE intercept changes. Wald test was used to determine which interaction terms to include in the final model with a significance threshold of 0.05. All final models included all sociodemographic, health-related, and digital access covariates. We reported pooled model outputs and generated pooled estimated marginal means (marginal effect at the mean) using ggeffects and plotted them using ggplot2.[4] Within each period, we conducted pairwise comparisons of the estimated marginal means using the emmeans package.[5]

---

[2] Buuren S van, Groothuis-Oudshoorn K. mice: Multivariate Imputation by Chained Equations in R. Journal of Statistical Software. 2011 Dec 12;45:1–67

[3] Fox J, Weisberg S (2019). *An R Companion to Applied Regression*, Third edition. Sage, Thousand Oaks CA. https://socialsciences.mcmaster.ca/jfox/Books/Companion/.

[4] Lüdecke D. ggeffects: Tidy Data Frames of Marginal Effects from Regression Models. Journal of Open Source Software. 2018 Jun 29;3(26):772.

[5] Lenth R (2022). _emmeans: Estimated Marginal Means, aka Least-Squares Means_. R package version 1.7.5, <https://CRAN.R-project.org/package=emmeans>.

© 2023 Nouri S et al. *JAMA Network Open.*

eTable 1. Encounter Types

Encounter type	Synchronous vs Asynchronous	Modality	Which clinicians?	Billed	Other notes
Outcome A: All encounters with any member of the team	Both	In-person, phone, video, or patient messages	Any member of the healthcare team	Mostly only for visits with billing clinician	Combination of Outcomes B + D
Outcome B: Visits with any member of the healthcare team	Synchronous	In-person, Phone, or Video	All members of the team (including billing clinicians)	Mostly only for visits with billing clinician	
Outcome C: Visits with billing clinicians	Synchronous	In-person, Phone, or Video	Billing clinicians	Yes, with rare exceptions	Subset of Outcome B
Outcome D: Between visit interactions	Both	Phone or patient messages	Any member of the healthcare team	No	Examples of phone calls and patient messages include requests to schedule or reschedule appointments, questions about billing, medication refill requests, clinical questions (e.g., a new symptom that is discussed over the phone with a nurse then discussed between the nurse and doctor).

© 2023 Nouri S et al. *JAMA Network Open.*

eTable 2. Interrupted Time-Series Model Coefficients at UCSF

	**UCSF Total Encounters n=4,967**	**UCSF Between-Visit Interactions n=4,967**	**UCSF Visits with Any Team Member n=4,967**	**UCSF Visits with Billing Clinicians n=4,967**
	Estimate[1] (std error)	Estimate (std error)	Estimate (std error)	Estimate (std error)
Intercept	**-0.267 (0.098)**	**-0.606 (0.109)**	**-1.492 (0.092)**	**-1.643 (0.082)**
Pre-PHE slope	-0.002 (0.002)	0.005 (0.003)	**-0.015 (0.002)**	**-0.017 (0.002)**
Pre-PHE intercept	Ref	Ref	Ref	Ref
PHE-SIP intercept change[2]	-0.023 (0.049)	-0.087 (0.063)	-0.010 (0.058)	0.003 (0.051)
PHE-SIP slope change	0.025 (0.016)	0.032 (0.017)	0.003 (0.020)	0.009 (0.020)
PHE-SIP intercept	Ref	Ref	Ref	Ref
Hybrid-PHE intercept change[2]	**-0.213 (0.054)**	**-0.237 (0.065)**	-0.063 (0.047)	-0.069 (0.038)
Hybrid-PHE slope change	-0.001 (0.016)	-0.007 (0.017)	0.011 (0.020)	0.006 (0.020)
Baseline BP <= 120/80	Ref	Ref	Ref	Ref
Baseline BP <=140/90	-0.045 (0.039)	-0.038 (0.043)	-0.033 (0.040)	0.020 (0.032)
Baseline BP >140/90	-0.046 (0.043)	-0.045 (0.046)	-0.041 (0.047)	0.009 (0.037)
Baseline A1c	**0.057 (0.012)**	**0.062 (0.012)**	**0.044 (0.011)**	**0.054 (0.009)**
Medicare	Ref	Ref	Ref	Ref
Commercial Insurance	**-0.247 (0.035)**	**-0.270 (0.041)**	**-0.164 (0.032)**	**-0.167 (0.029)**
Medicaid	0.021 (0.049)	0.019 (0.055)	0.055 (0.054)	0.023 (0.043)
Charlson Comorbidity Index 0-2	Ref	Ref	Ref	Ref
Charlson Comorbidity Index 3+	**0.518 (0.030)**	**0.506 (0.034)**	**0.511 (0.034)**	**0.453 (0.026)**
Patient Portal Activated	Ref	Ref	Ref	Ref
Patient Portal Not Activated	**-0.200 (0.038)**	**-0.276 (0.044)**	-0.054 (0.037)	**-0.070 (0.029)**
Male	Ref	Ref	Ref	Ref
Female	**0.203 (0.028)**	**0.206 (0.031)**	**0.185 (0.027)**	**0.162 (0.023)**
nSES quintile 5	Ref	Ref	Ref	Ref
nSES quintile 4	0.005 (0.045)	0.010 (0.051)	-0.002 (0.039)	0.000 (0.037)
nSES quintile 3	-0.006 (0.045)	-0.007 (0.050)	-0.016 (0.042)	-0.006 (0.038)
nSES quintile 2	0.044 (0.046)	0.037 (0.051)	0.064 (0.047)	0.048 (0.039)
nSES quintile 1	0.090 (0.051)	0.100 (0.057)	0.072 (0.045)	**0.088 (0.042)**
English Language	Ref	Ref	Ref	Ref
Chinese Language	-0.049 (0.054)	**-0.195 (0.063)**	0.050 (0.045)	0.052 (0.042)
Spanish Language	0.014 (0.086)	0.005 (0.089)	0.092 (0.071)	0.119 (0.068)
Other/Unknown Language	-0.085 (0.059)	**-0.200 (0.062)**	0.027 (0.047)	0.041 (0.043)
Age 75+	Ref	Ref	Ref	Ref
Age 18-34	**-0.230 (0.097)**	**-0.268 (0.107)**	-0.199 (0.113)	**-0.301 (0.087)**
Age 35-49	**-0.132 (0.062)**	**-0.189 (0.073)**	-0.045 (0.062)	**-0.106 (0.049)**
Age 50-64	-0.030 (0.048)	-0.074 (0.059)	0.038 (0.038)	-0.042 (0.036)
Age 65-74	-0.039 (0.039)	-0.062 (0.049)	-0.005 (0.032)	-0.059 (0.031)
NH White	Ref	Ref	Ref	Ref
NH Asian	**-0.245 (0.042)**	**-0.322 (0.051)**	-0.026 (0.035)	0.004 (0.033)
NH Black or African American	**0.147 (0.053)**	0.113 (0.066)	**0.205 (0.050)**	**0.179 (0.044)**
Hispanic or Latino	-0.020 (0.057)	-0.055 (0.071)	0.051 (0.050)	0.063 (0.047)
Other/Unknown Race/Ethnicity	**-0.154 (0.056)**	**-0.197 (0.065)**	-0.033 (0.059)	-0.023 (0.044)
**Interaction Terms**				
Age 18-34: PHE-SIP intercept change	0.081 (0.130)	0.110 (0.142)	0.075 (0.153)	
Age 35-49: PHE-SIP intercept change	0.089 (0.067)	0.120 (0.075)	0.077 (0.068)	
Age 50-64: PHE-SIP intercept change	0.035 (0.050)	0.055 (0.057)	0.039 (0.049)	
Age 65-74: PHE-SIP intercept change	-0.057 (0.051)	-0.036 (0.057)	-0.062 (0.051)	
Age 18-34: Hybrid-PHE intercept change	0.117 (0.127)	0.136 (0.137)	0.060 (0.163)	0.158 (0.102)
Age 35-49: Hybrid-PHE intercept change	0.116 (0.070)	0.133 (0.077)	0.084 (0.071)	0.082 (0.050)
Age 50-64: Hybrid-PHE intercept change	0.081 (0.053)	0.097 (0.061)	0.045 (0.051)	0.052 (0.037)

© 2023 Nouri S et al. *JAMA Network Open.*

	UCSF Total Encounters n=4,967	UCSF Between-Visit Interactions n=4,967	UCSF Visits with Any Team Member n=4,967	UCSF Visits with Billing Clinicians n=4,967
Age 65-74: Hybrid-PHE intercept change	**0.113 (0.054)**	0.111 (0.061)	**0.122 (0.052)**	**0.081 (0.036)**
NH Asian: Hybrid-PHE intercept change	**0.145 (0.040)**	**0.132 (0.058)**		
NH Black or African American: Hybrid-PHE intercept change	0.023 (0.050)	-0.008 (0.063)		
Hispanic or Latino: Hybrid-PHE intercept change	0.022 (0.054)	0.016 (0.068)		
Other/Unknown Race/Ethnicity: Hybrid-PHE intercept change	**0.149 (0.056)**	**0.163 (0.081)**		
NH Asian: PHE-SIP intercept change		0.040 (0.053)	-0.004 (0.039)	-0.010 (0.036)
NH Black or African American: PHE-SIP intercept change		0.030 (0.063)	0.086 (0.047)	0.061 (0.043)
Hispanic or Latino: PHE-SIP intercept change		0.030 (0.070)	-0.010 (0.055)	0.018 (0.050)
Other/Unknown Race/Ethnicity: PHE-SIP intercept change		-0.024 (0.081)	**0.141 (0.060)**	0.063 (0.052)
Chinese Language: PHE-SIP intercept change	**-0.160 (0.054)**		**-0.202 (0.072)**	**-0.116 (0.049)**
Spanish Language: PHE-SIP intercept change	0.020 (0.084)		-0.002 (0.106)	-0.010 (0.078)
Other/Unknown Language: PHE-SIP intercept change	**-0.127 (0.056)**		**-0.170 (0.081)**	**-0.108 (0.049)**
Chinese Language: Hybrid-PHE intercept change			0.073 (0.072)	
Spanish Language: Hybrid-PHE intercept change			0.026 (0.103)	
Other/Unknown Language: Hybrid-PHE intercept change			0.037 (0.085)	

[1]Poisson regression coefficients on the log scale.
[2]Given the interaction terms included in the models, these intercept changes represent the intercept change when covariates are at their reference level (those ages 75+, English speakers, NH White)
Significant estimates (p<0.05) are bolded. Standard errors are robust.
Multiple imputation by chained equations (MICE) procedure used for missing values for baseline A1c and baseline blood pressure.

© 2023 Nouri S et al. *JAMA Network Open.*

eTable 3. Interrupted Time-Series Model Coefficients at SFHN

	SFHN Total Encounters n=8,975	SFHN Between-Visit Interactions n=8,975	SFHN Visits with Any Team Member n=8,975	SFHN Visits with Billing Clinicians n=8,975
	Estimate[1] (std error)	Estimate (std error)	Estimate (std error)	Estimate (std error)
Intercept	-0.146 (0.082)	**-0.462 (0.102)**	**-1.206 (0.089)**	**-1.445 (0.081)**
Pre-PHE slope	**0.031 (0.003)**	**0.048 (0.004)**	**0.009 (0.004)**	**0.010 (0.004)**
Pre-PHE intercept	Ref	Ref	Ref	Ref
PHE-SIP intercept change[2]	-0.012 (0.049)	0.023 (0.071)	**-0.170 (0.068)**	-0.073 (0.070)
PHE-SIP slope change	**-0.057 (0.011)**	**-0.105 (0.013)**	0.013 (0.013)	0.004 (0.014)
PHE-SIP intercept	Ref	Ref	Ref	Ref
Hybrid-PHE intercept change[2]	**-0.120 (0.036)**	**-0.168 (0.044)**	-0.057 (0.064)	-0.112 (0.062)
Hybrid-PHE slope change	**0.044 (0.011)**	**0.085 (0.013)**	-0.020 (0.013)	-0.013 (0.014)
Baseline BP <= 120/80	Ref	Ref	Ref	Ref
Baseline BP <=140/90	0.025 (0.024)	0.037 (0.028)	0.011 (0.027)	0.032 (0.023)
Baseline BP >140/90	-0.002 (0.030)	0.010 (0.035)	-0.011 (0.034)	-0.003 (0.029)
Baseline A1c	**0.056 (0.006)**	**0.045 (0.007)**	**0.058 (0.007)**	**0.047 (0.007)**
Medicare	Ref	Ref	Ref	Ref
Commercial Insurance	**-0.700 (0.090)**	**-0.667 (0.096)**	**-0.726 (0.103)**	**-0.737 (0.098)**
Medicaid	**-0.054 (0.027)**	**-0.083 (0.031)**	-0.005 (0.030)	-0.013 (0.025)
Uninsured	**-0.313 (0.040)**	**-0.419 (0.049)**	**-0.174 (0.043)**	**-0.169 (0.039)**
Healthy Workers	**-0.285 (0.031)**	**-0.375 (0.036)**	**-0.161 (0.035)**	**-0.139 (0.030)**
Charlson Comorbidity Index 0-2	Ref	Ref	Ref	Ref
Charlson Comorbidity Index 3+	**0.379 (0.026)**	**0.385 (0.031)**	**0.366 (0.028)**	**0.370 (0.024)**
Patient Portal Activated	Ref	Ref	Ref	Ref
Patient Portal Not Activated	**-0.456 (0.032)**	**-0.533 (0.037)**	**-0.332 (0.034)**	**-0.306 (0.029)**
Male	Ref	Ref	Ref	Ref
Female	**0.141 (0.020)**	**0.180 (0.024)**	**0.081 (0.022)**	**0.114 (0.019)**
nSES quintile 5	Ref	Ref	Ref	Ref
nSES quintile 4	-0.007 (0.050)	-0.069 (0.065)	0.028 (0.046)	-0.011 (0.043)
nSES quintile 3	0.007 (0.049)	-0.109 (0.063)	0.055 (0.048)	0.026 (0.043)
nSES quintile 2	-0.011 (0.047)	-0.093 (0.061)	0.050 (0.043)	0.010 (0.041)
nSES quintile 1	0.043 (0.046)	-0.061 (0.059)	**0.132 (0.043)**	0.066 (0.040)
English Language	Ref	Ref	Ref	Ref
Chinese Language	**-0.163 (0.039)**	**-0.183 (0.047)**	**-0.133 (0.040)**	**-0.161 (0.038)**
Spanish Language	**-0.102 (0.045)**	**-0.284 (0.057)**	**0.140 (0.046)**	0.078 (0.044)
Other/Unknown Language	-0.061 (0.038)	**-0.099 (0.048)**	-0.004 (0.041)	0.039 (0.038)
Age 75+	Ref	Ref	Ref	Ref
Age 18-34	**-0.337 (0.081)**	**-0.366 (0.096)**	**-0.289 (0.090)**	**-0.302 (0.085)**
Age 35-49	**-0.141 (0.049)**	**-0.130 (0.058)**	**-0.165 (0.055)**	**-0.193 (0.047)**
Age 50-64	0.000 (0.037)	-0.024 (0.046)	0.029 (0.042)	-0.028 (0.036)
Age 65-74	0.010 (0.035)	0.014 (0.044)	0.003 (0.039)	-0.034 (0.033)
NH White	Ref	Ref	Ref	Ref
NH Asian	**-0.282 (0.042)**	**-0.353 (0.051)**	**-0.172 (0.047)**	**-0.103 (0.042)**
NH Black or African American	0.064 (0.042)	0.069 (0.051)	0.056 (0.054)	0.030 (0.043)
Hispanic or Latino	-0.094 (0.051)	-0.089 (0.063)	-0.094 (0.056)	-0.001 (0.051)
Other/Unknown Race/Ethnicity	-0.098 (0.057)	-0.048 (0.069)	**-0.173 (0.065)**	-0.102 (0.057)
**Interaction Terms**				
Chinese Language: PHE-SIP intercept change	-0.069 (0.050)	**-0.161 (0.060)**	0.065 (0.057)	0.080 (0.059)
Spanish Language: PHE-SIP intercept change	-0.008 (0.050)	0.072 (0.061)	-0.087 (0.059)	-0.077 (0.060)
Other/Unknown Language: PHE-SIP intercept change	**-0.124 (0.050)**	-0.115 (0.059)	**-0.131 (0.059)**	**-0.129 (0.060)**

© 2023 Nouri S et al. *JAMA Network Open.*

	SFHN Total Encounters n=8,975	SFHN Between-Visit Interactions n=8,975	SFHN Visits with Any Team Member n=8,975	SFHN Visits with Billing Clinicians n=8,975
Age 18-34: PHE-SIP intercept change	**0.212 (0.078)**	**0.242 (0.089)**	0.198 (0.113)	0.221 (0.113)
Age 35-49: PHE-SIP intercept change	**0.142 (0.042)**	0.074 (0.051)	**0.266 (0.062)**	**0.239 (0.060)**
Age 50-64: PHE-SIP intercept change	**0.070 (0.035)**	0.049 (0.043)	0.092 (0.049)	0.052 (0.050)
Age 65-74: PHE-SIP intercept change	0.032 (0.036)	0.020 (0.045)	0.064 (0.053)	0.033 (0.052)
NH Asian: PHE-SIP intercept change	0.045 (0.048)	0.076 (0.058)	-0.000 (0.060)	-0.082 (0.061)
NH Black or African American: PHE-SIP intercept change	**0.102 (0.044)**	0.090 (0.052)	0.116 (0.060)	0.086 (0.058)
Hispanic or Latino: PHE-SIP intercept change	**0.142 (0.056)**	0.131 (0.067)	**0.158 (0.069)**	0.095 (0.069)
Other/Unknown Race/Ethnicity: PHE-SIP intercept change	0.060 (0.066)	-0.018 (0.081)	**0.180 (0.076)**	0.090 (0.078)
Chinese Language: Hybrid-PHE intercept change	**0.125 (0.049)**	**0.187 (0.058)**	0.035 (0.056)	0.052 (0.057)
Spanish Language: Hybrid-PHE intercept change	0.078 (0.050)	0.070 (0.061)	0.080 (0.055)	**0.117 (0.059)**
Other/Unknown Language: Hybrid-PHE intercept change	**0.233 (0.050)**	**0.259 (0.059)**	**0.189 (0.058)**	**0.166 (0.059)**
NH Asian: Hybrid-PHE intercept change	0.019 (0.048)	0.026 (0.057)	0.010 (0.058)	0.039 (0.058)
NH Black or African American: Hybrid-PHE intercept change	-0.053 (0.045)	-0.038 (0.052)	-0.080 (0.061)	-0.031 (0.055)
Hispanic or Latino: Hybrid-PHE intercept change	0.002 (0.056)	0.002 (0.068)	-0.000 (0.065)	0.027 (0.067)
Other/Unknown Race/Ethnicity: Hybrid-PHE intercept change	-0.022 (0.063)	0.017 (0.076)	-0.087 (0.073)	0.037 (0.073)
nSES quintile 4: PHE-SIP intercept change		0.043 (0.058)		
nSES quintile 3: PHE-SIP intercept change		**0.111 (0.055)**		
nSES quintile 2: PHE-SIP intercept change		0.052 (0.053)		
nSES quintile 1: PHE-SIP intercept change		0.059 (0.052)		
Age 18-34: Hybrid-PHE intercept change			-0.027 (0.109)	-0.062 (0.107)
Age 35-49: Hybrid-PHE intercept change			-0.011 (0.067)	-0.025 (0.058)
Age 50-64: Hybrid-PHE intercept change			0.035 (0.049)	0.045 (0.048)
Age 65-74: Hybrid-PHE intercept change			-0.012 (0.051)	-0.007 (0.050)

[1]Poisson regression coefficients on the log scale.
[2]Given the interaction terms included in the models, these intercept changes represent the intercept change when covariates are at their reference level (those ages 75+, English speakers, NH White)
Significant estimates (p<0.05) are bolded. Standard errors are robust.
Multiple imputation by chained equations (MICE) procedure used for missing values for baseline A1c and baseline blood pressure.

© 2023 Nouri S et al. *JAMA Network Open.*

eTable 4. Slope and Slope Change Incident Rate Ratios

	**UCSF Total Encounters n=4,967**	**UCSF Between-Visit Interactions n=4,967**	**UCSF Visits with Any Team Member n=4,967**	**UCSF Visits with Billing Clinicians n=4,967**
	IRR (95% CI)	IRR (95% CI)	IRR (95% CI)	IRR (95% CI)
Pre-PHE slope	0.998 (0.994, 1.003)	1.005 (0.999, 1.010)	**0.985 (0.981, 0.989)**	**0.984 (0.980, 0.988)**
PHE-SIP slope	1.024 (0.994, 1.055)	**1.038 (1.004, 1.072)**	0.988 (0.950, 1.028)	0.992 (0.954, 1.032)
Hybrid-PHE slope	**1.023 (1.016, 1.029)**	**1.031 (1.023, 1.038)**	0.999 (0.992, 1.006)	0.999 (0.991, 1.006)
Slope change from Pre-PHE to Hybrid-PHE	**1.024 (1.016, 1.032)**	**1.026 (1.017, 1.036)**	**1.014 (1.006, 1.023)**	**1.015 (1.007, 1.023)**
	**SFHN Total Encounters n=8,975**	**SFHN Between-Visit Interactions n=8,975**	**SFHN Visits with Any Team Member n=8,975**	**SFHN Visits with Billing Clinicians n=8,975**
	IRR (95% CI)	IRR (95% CI)	IRR (95% CI)	IRR (95% CI)
Pre-PHE slope	**1.031 (1.025, 1.038)**	**1.049 (1.041, 1.057)**	**1.009 (1.001, 1.016)**	**1.010 (1.003, 1.017)**
PHE-SIP slope	**0.974 (0.955, 0.994)**	0.945 (0.922, 0.968)	1.022 (0.997, 1.048)	1.014 (0.987, 1.042)
Hybrid-PHE slope	**1.018 (1.013, 1.022)**	**1.029 (1.023, 1.034)**	1.002 (0.996, 1.007)	1.001 (0.995, 1.006)
Slope change from Pre-PHE to Hybrid-PHE	**0.987 (0.979, 0.995)**	**0.981 (0.972 0.990)**	0.993 (0.984, 1.002)	**0.991 (0.982, 0.999)**

IRR = Incident rate ratio. This is determined using the equations below and describes the rate at which encounters are increasing/decreasing each month. For example, Pre-PHE slope for UCSF encounters = 0.998, which indicates a decrease of (1-0.998 = 0.2%) each month in that period. Similarly, the slope change from pre-PHE to hybrid-PHE for UCSF total encounters = 1.024, which is interpreted as an increase in slope of 2.4%.

Statistically significant values are bolded ($p<0.05$).

IRRs and 95% CIs were calculated using model coefficients in eTable 2 and eTable 3:
Pre-PHE slope IRR = exp(Pre-PHE slope)
Hybrid-PHE slope IRR = exp(Pre-PHE slope + PHE-SIP slope change + Hybrid-PHE slope change)
Slope change from Pre-PHE to Hybrid-PHE IRR= exp(Pre-PHE slope + PHE-SIP slope change + Hybrid-PHE slope change – Pre-PHE slope)which is mathematically equivalent to Hybrid-PHE slope IRR/Pre-PHE slope IRR

© 2023 Nouri S et al. *JAMA Network Open.*

eTable 5. Estimated Marginal Means

System	Encounter Type	Age<br>Mean (95% CI)				Race/Ethnicity<br>Mean (95% CI)				Language<br>Mean (95% CI)			
			Pre	SIP	Hybrid		Pre	SIP	Hybrid		Pre	SIP	Hybrid
UCSF	Total Encounters	18-34	1.07 (0.88, 1.30)	1.08 (0.93, 1.41)	1.04 (0.85, 1.28)	White	1.31 (1.18, 1.46)	1.25 (1.12, 1.40)	1.09 (0.97, 1.22)	English	1.28 (1.19, 1.38)	1.31 (1.21, 1.42)	1.22 (1.13, 1.32)
		35-49	1.19 (1.05, 1.35)	1.20 (1.04, 1.39)	1.16 (1.02, 1.31)	Asian	1.02 (0.93, 1.13)	0.98 (0.88, 1.09)	0.99 (0.89, 1.09)	Chinese	1.22 (1.08, 1.38)	1.06 (0.92, 1.22)	0.99 (0.86, 1.14)
		50-64	1.31 (1.20, 1.44)	1.26 (1.14, 1.40)	1.17 (1.06, 1.30)	Black	1.52 (1.35, 1.71)	1.45 (1.28, 1.64)	1.29 (1.14, 1.46)	Spanish	1.29 (1.09, 1.55)	1.35 (1.12, 1.63)	1.26 (1.05, 1.52)
		65-74	1.30 (1.19, 1.43)	1.14 (1.03, 1.27)	1.09 (0.99, 1.21)	Hispanic	1.29 (1.16, 1.43)	1.23 (1.09, 1.38)	1.09 (0.97, 1.23)	Other	1.18 (1.04, 1.34)	1.06 (0.92, 1.21)	0.99 (0.86, 1.12)
		75+	1.36 (1.24, 1.48)	1.26 (1.12, 1.41)	1.07 (0.97, 1.19)	Other	1.13 (1.00, 1.27)	1.07 (0.95, 1.22)	1.09 (0.95, 1.25)				
	Visits with Billing Clinicians	18-34	0.25 (0.21, 0.30)	0.28 (0.23, 0.33)	0.27 (0.22, 0.34)	White	0.29 (0.27, 0.32)	0.32 (0.28, 0.35)	0.29 (0.25, 0.32)	English	0.29 (0.27, 0.31)	0.34 (0.32, 0.37)	0.31 (0.28, 0.34)
		35-49	0.30 (0.27, 0.34)	0.34 (0.30, 0.38)	0.31 (0.27, 0.35)	Asian	0.29 (0.27, 0.32)	0.31 (0.29, 0.35)	0.28 (0.26, 0.32)	Chinese	0.31 (0.28, 0.34)	0.32 (0.28, 0.36)	0.29 (0.25, 0.33)
		50-64	0.33 (0.30, 0.35)	0.36 (0.33, 0.40)	0.32 (0.29, 0.36)	Black	0.35 (0.32, 0.39)	0.40 (0.36, 0.45)	0.36 (0.32, 0.41)	Spanish	0.33 (0.28, 0.38)	0.38 (0.32, 0.46)	0.34 (0.28, 0.42)
		65-74	0.32 (0.30, 0.35)	0.36 (0.32, 0.39)	0.32 (0.29, 0.36)	Hispanic	0.31 (0.28, 0.34)	0.34 (0.31, 0.38)	0.31 (0.28, 0.35)	Other	0.30 (0.27, 0.34)	0.32 (0.28, 0.36)	0.29 (0.25, 0.33)
		75+	0.34 (0.31, 0.37)	0.38 (0.34, 0.41)	0.31 (0.28, 0.35)	Other	0.29 (0.26, 0.32)	0.33 (0.29, 0.37)	0.30 (0.26, 0.34)				
	Visits with any team member	18-34	0.31 (0.24, 0.38)	0.35 (0.26, 0.47)	0.33 (0.26, 0.41)	White	0.34 (0.31, 0.38)	0.36 (0.32, 0.41)	0.34 (0.30, 0.38)	English	0.34 (0.31, 0.38)	0.41 (0.37, 0.45)	0.37 (0.34, 0.41)
		35-49	0.36 (0.31, 0.41)	0.41 (0.34, 0.49)	0.39 (0.33, 0.47)	Asian	0.34 (0.30, 0.37)	0.35 (0.31, 0.39)	0.33 (0.29, 0.37)	Chinese	0.36 (0.32, 0.41)	0.35 (0.30, 0.42)	0.34 (0.30, 0.40)
		50-64	0.39 (0.35, 0.43)	0.43 (0.38, 0.49)	0.39 (0.33, 0.47)	Black	0.42 (0.37, 0.48)	0.48 (0.41, 0.56)	0.45 (0.39, 0.53)	Spanish	0.38 (0.32, 0.44)	0.45 (0.36, 0.57)	0.42 (0.34, 0.52)
		65-74	0.37 (0.34, 0.41)	0.37 (0.33, 0.42)	0.37 (0.33, 0.41)	Hispanic	0.36 (0.33, 0.40)	0.38 (0.33, 0.43)	0.35 (0.31, 0.40)	Other	0.35 (0.32, 0.39)	0.36 (0.29, 0.43)	0.34 (0.29, 0.39)
		75+	0.37 (0.34, 0.41)	0.40 (0.35, 0.45)	0.35 (0.31, 0.39)	Other	0.33 (0.29, 0.38)	0.40 (0.33, 0.48)	0.38 (0.31, 0.45)				
	Between-visit interactions	18-34	0.73 (0.59, 0.90)	0.73 (0.54, 0.98)	0.72 (0.66, 0.82)	White	0.93 (0.82, 1.04)	0.86 (0.75, 0.98)	0.77 (0.68, 0.87)	English	0.93 (0.86, 1.01)	0.88 (0.80, 0.96)	0.83 (0.77, 0.91)
		35-49	0.79 (0.68, 0.91)	0.80, (0.68, 0.93)	0.79 (0.69, 0.90)	Asian	0.67 (0.60, 0.75)	0.65 (0.57, 0.74)	0.66 (0.60, 0.74)	Chinese	0.77 (0.67, 0.88)	0.72 (0.62, 0.83)	0.69 (0.60, 0.79)
		50-64	0.88 (0.79, 0.98)	0.84 (0.75, 0.94)	0.80 (0.71, 0.89)	Black	1.04 (0.90, 1.19)	0.99 (0.86, 1.15)	0.88 (0.77, 1.01)	Spanish	0.93 (0.78, 1.12)	0.88 (0.73, 1.06)	0.84 (0.70, 1.01)
		65-74	0.89 (0.80, 0.99)	0.77 (0.69, 0.86)	0.80 (0.71, 0.89)	Hispanic	0.88 (0.77, 0.99)	0.84 (0.72, 0.97)	0.76 (0.67, 0.86)	Other	0.76 (0.66, 0.87)	0.72 (0.62, 0.83)	0.68 (0.59, 0.78)
		75+	0.95 (0.86, 1.05)	0.85 (0.75, 0.97)	0.74 (0.66, 0.82)	Other	0.76 (0.66, 0.87)	0.69 (0.58, 0.82)	0.72 (0.63, 0.83)				
SFHN	Total Encounters	18-34	0.84 (0.72, 0.98)	1.06 (0.90, 1.24)	0.86 (0.73, 1.01)	White	1.16 (1.06, 1.28)	1.21 (1.09, 1.34)	0.99 (0.90, 1.09)	English	1.16 (1.08, 1.25)	1.37 (1.27, 1.46)	0.99 (0.93, 1.07)
		35-49	1.02 (0.93, 1.12)	1.20 (1.10, 1.31)	0.98 (0.90, 1.06)	Asian	0.88 (0.80, 0.95)	0.96 (0.88, 1.04)	0.80 (0.74, 0.87)	Chinese	0.99 (0.89, 1.09)	1.08 (0.97, 1.21)	0.89 (0.81, 0.98)
		50-64	1.18 (1.09, 1.27)	1.29 (1.20, 1.38)	1.05 (0.97, 1.12)	Black	1.24 (1.12, 1.36)	1.43 (1.29, 1.58)	1.11 (1.02, 1.22)	Spanish	1.05 (0.94, 1.17)	1.22 (1.10, 1.36)	0.96 (0.87, 1.07)
		65-74	1.19 (1.09, 1.29)	1.25 (1.16, 1.35)	1.02 (0.94, 1.10)	Hispanic	1.06 (0.96, 1.16)	1.27 (1.16, 1.40)	1.05 (0.96, 1.14)	Other	1.09 (0.99, 1.20)	1.13 (1.01, 1.27)	1.04 (0.95, 1.15)
		75+	1.17 (1.07, 1.29)	1.20 (1.09, 1.31)	0.97 (0.89, 1.07)	Other	1.05 (0.94, 1.19)	1.17 (1.02, 1.34)	0.94 (9.83, 1.05)				
	Visits with Billing Clinicians	18-34	0.24 (0.21, 0.29)	0.29 (0.23, 0.36)	0.25 (0.21, 0.29)	White	0.31 (0.27, 0.34)	0.31 (0.27, 0.35)	0.28 (0.25, 0.31)	English	0.29 (0.27, 0.32)	0.32 (0.30, 0.35)	0.27 (0.25, 0.30)
		35-49	0.27 (0.24, 0.31)	0.32 (0.29, 0.36)	0.29 (0.27, 0.33)	Asian	0.28 (0.25, 0.30)	0.26 (0.23, 0.28)	0.24 (0.22, 0.26)	Chinese	0.25 (0.23, 0.28)	0.30 (0.26, 0.34)	0.27 (0.24, 0.29)
		50-64	0.32 (0.29, 0.35)	0.32 (0.29, 0.34)	0.31 (0.29, 0.34)	Black	0.31 (0.28, 0.35)	0.35 (0.31, 0.39)	0.30 (0.28, 0.34)	Spanish	0.32 (0.29, 0.36)	0.32 (0.29, 0.36)	0.31 (0.28, 0.34)
		65-74	0.32 (0.29, 0.35)	0.31 (0.28, 0.34)	0.29 (0.26, 0.31)	Hispanic	0.30 (0.28, 0.34)	0.34 (0.30. 0.38)	0.32 (0.29, 0.35)	Other	0.31 (0.28, 0.35)	0.29 (0.26, 0.33)	0.29 (0.27, 0.33)
		75+	0.33 (0.30, 0.36)	0.31 (0.28, 0.35)	0.29 (0.26, 0.32)	Other	0.28 (0.24, 0.31)	0.30 (0.26, 0.35)	0.29 (0.25, 0.32)				
	Visits with any team member	18-34	0.33 (0.28, 0.40)	0.36 (0.29, 0.45)	0.33 (0.28, 0.40)	White	0.44 (0.39, 0.49)	0.41 (0.36, 0.46)	0.39 (0.35, 0.44)	English	0.41 (0.37, 0.44)	0.43 (0.39, 0.46)	0.37 (0.34, 0.40)
		35-49	0.38 (0.34, 0.42)	0.44 (0.39, 0.50)	0.41 (0.37, 0.45)	Asian	0.37 (0.34, 0.41)	0.34 (0.31, 0.38)	0.33 (0.30, 0.37)	Chinese	0.36 (0.32, 0.40)	0.40 (0.35, 0.45)	0.36 (0.32, 0.40)
		50-64	0.46 (0.42. 0.49)	0.45 (0.41, 0.49)	0.43 (0.40, 0.47)	Black	0.46 (0.41, 0.52)	0.48 (0.42, 0.55)	0.43 (0.39, 0.48)	Spanish	0.47 (0.42, 0.52)	0.45 (0.40, 0.51)	0.42 (0.38, 0.47)
		65-74	0.44 (0.41, 0.49)	0.42 (0.39, 0.46)	0.39 (0.36, 0.43)	Hispanic	0.40 (0.36, 0.44)	0.43 (0.39, 0.48)	0.42 (0.38, 0.46)	Other	0.41 (0.37, 0.45)	0.37 (0.33, 0.43)	0.39 (0.35, 0.43)
		75+	0.44 (0.40, 0.49)	0.40 (0.35, 0.44)	0.37 (0.33, 0.41)	Other	0.37 (0.32, 0.42)	0.41 (0.35, 0.47)	0.36 (0.32, 0.41)				
	Between-visit interactions	18-34	0.51 (0.42, 0.61)	0.72 (0.59, 0.87)	0.52 (0.43, 0.63)	White	0.72 (0.64, 0.81)	0.81 (0.72, 0.92)	0.59 (0.53, 0.66)	English	0.76 (0.70, 0.83)	0.96 (0.89, 1.04)	0.61 (0.56, 0.66)
		35-49	0.64 (0.58, 0.72)	0.77 (0.69, 0.85)	0.55 (0.50, 0.61)	Asian	0.51 (0.46, 0.56)	0.62 (0.56, 0.68)	0.46 (0.42, 0.50)	Chinese	0.64 (0.56, 0.71)	0.68 (0.60. 0.77)	0.52 (0.47, 0.58)
		50-64	0.72 (0.66, 0.78)	0.83 (0.77, 0.90)	0.60 (0.55, 0.65)	Black	0.77 (0.69, 0.87)	0.95 (0.85, 1.07)	0.66 (0.60, 0.74)	Spanish	0.57 (0.50, 0.65)	0.78 (0.68, 0.88)	0.53 (0.47, 0.60)
		65-74	0.74 (0.67, 0.82)	0.84 (0.77, 0.92)	0.61 (0.55, 0.66)	Hispanic	0.66 (0.59. 0.74)	0.85 (0.76, 0.94)	0.62 (0.55, 0.68)	Other	0.69 (0.61, 0.78)	0.77 (0.68, 0.88)	0.64 (0.57, 0.71)
		75+	0.73 (0.65, 0.82)	0.81 (0.73, 0.90)	0.59 (0.53, 0.65)	Other	0.69 (0.59, 0.79)	0.76 (0.65, 0.90)	0.56 (0.49, 0.64)				

© 2023 Nouri S et al. *JAMA Network Open.*

eFigure 1. Interrupted Time-Series Analysis of Patient-Clinician Encounters per Month at UCSF (n = 4967) and SFHN (n = 8975)

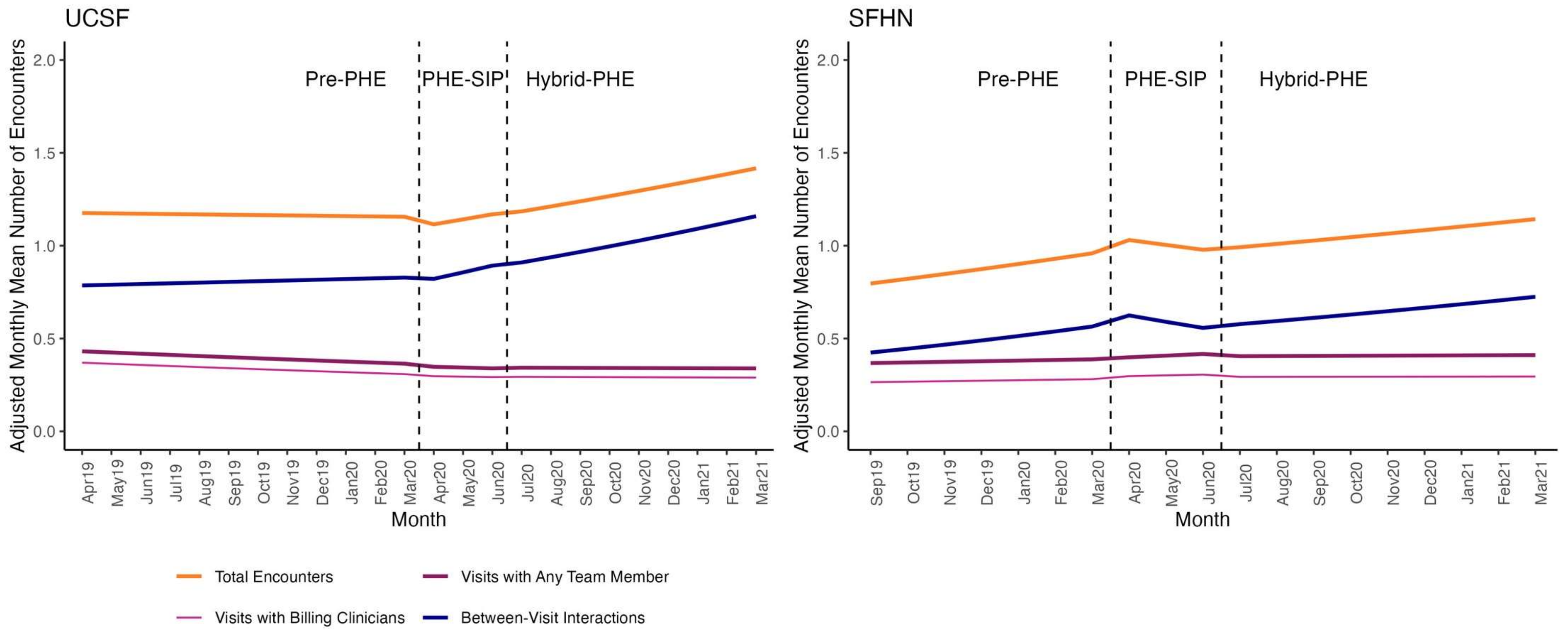

© 2023 Nouri S et al. *JAMA Network Open.*

eFigure 2. Interrupted Time-Series Analysis of Patient-Clinician Encounters per Month at UCSF (n = 4967) Stratified by Patient Age

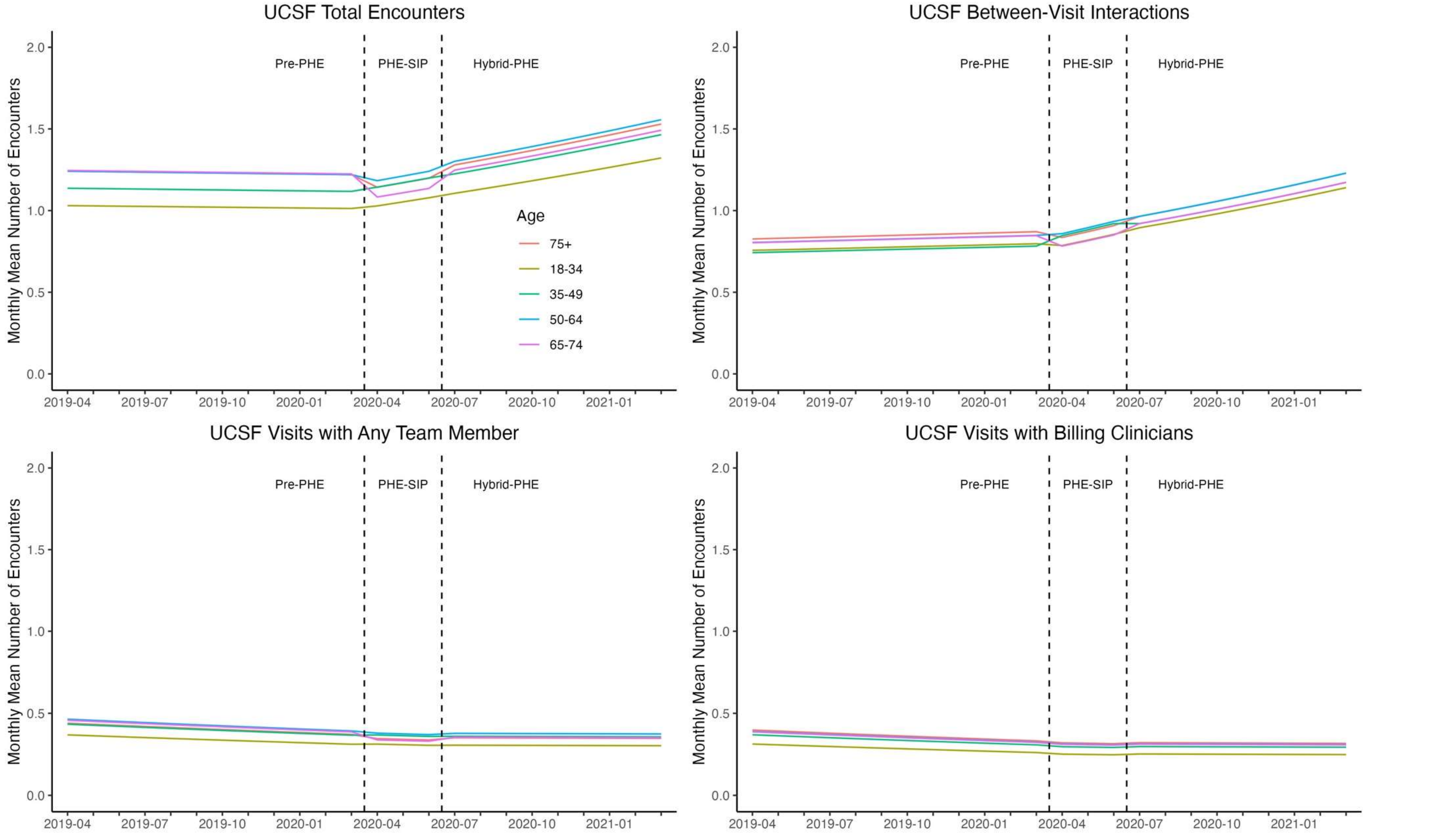

Source Authors' analysis of electronic health record data.

Notes Results are adjusted for sex, race/ethnicity, language, neighborhood socioeconomic status, health insurance type, Charlson co-morbidity index, baseline hemoglobin A1c, baseline blood pressure, and enrollment in the patient portal. Pre-PHE = Pre COVID-19 Public Health Emergency (4/1/2019-3/31/2020). PHE-SIP = Public Health Emergency Shelter in Place (4/1/2020-6/30/2020). Hybrid-PHE = Hybrid Public Health Emergency (7/1/2020-3/31/2021).

© 2023 Nouri S et al. *JAMA Network Open.*

eFigure 3. Interrupted Time-Series Analysis of Patient-Clinician Encounters per Month at SFHN (n = 8975) Stratified by Patient Age

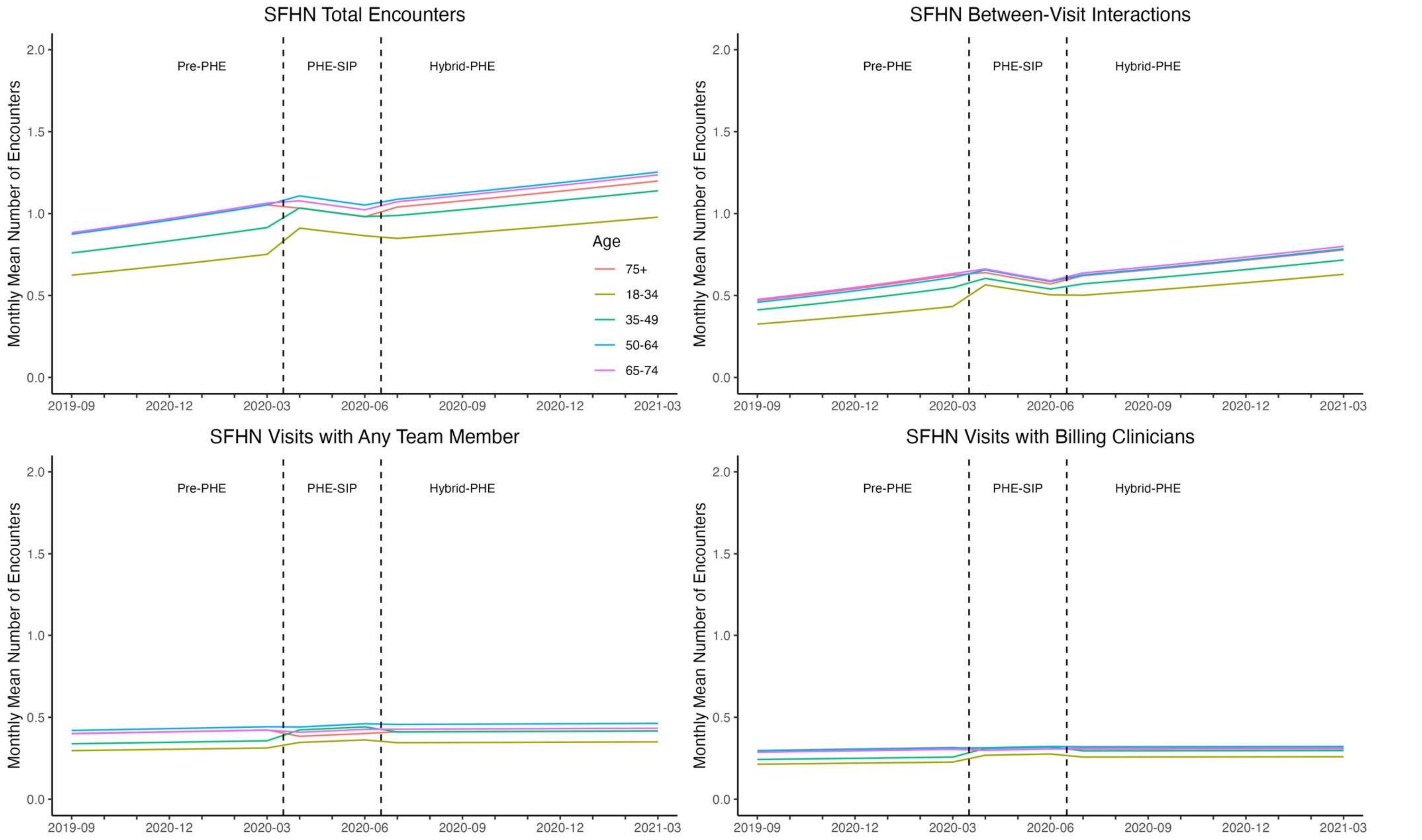

Source Authors' analysis of electronic health record data.

Notes Results are adjusted for sex, race/ethnicity, language, neighborhood socioeconomic status, health insurance type, Charlson co-morbidity index, baseline hemoglobin A1c, baseline blood pressure, and enrollment in the patient portal. Pre-PHE = Pre COVID-19 Public Health Emergency (4/1/2019-3/31/2020). PHE-SIP = Public Health Emergency Shelter in Place (4/1/2020-6/30/2020). Hybrid-PHE = Hybrid Public Health Emergency (7/1/2020-3/31/2021).

© 2023 Nouri S et al. *JAMA Network Open.*

eFigure 4. Interrupted Time-Series Analysis of Patient-Clinician Encounters per Month at UCSF (n = 4967) Stratified by Patient Race and Ethnicity

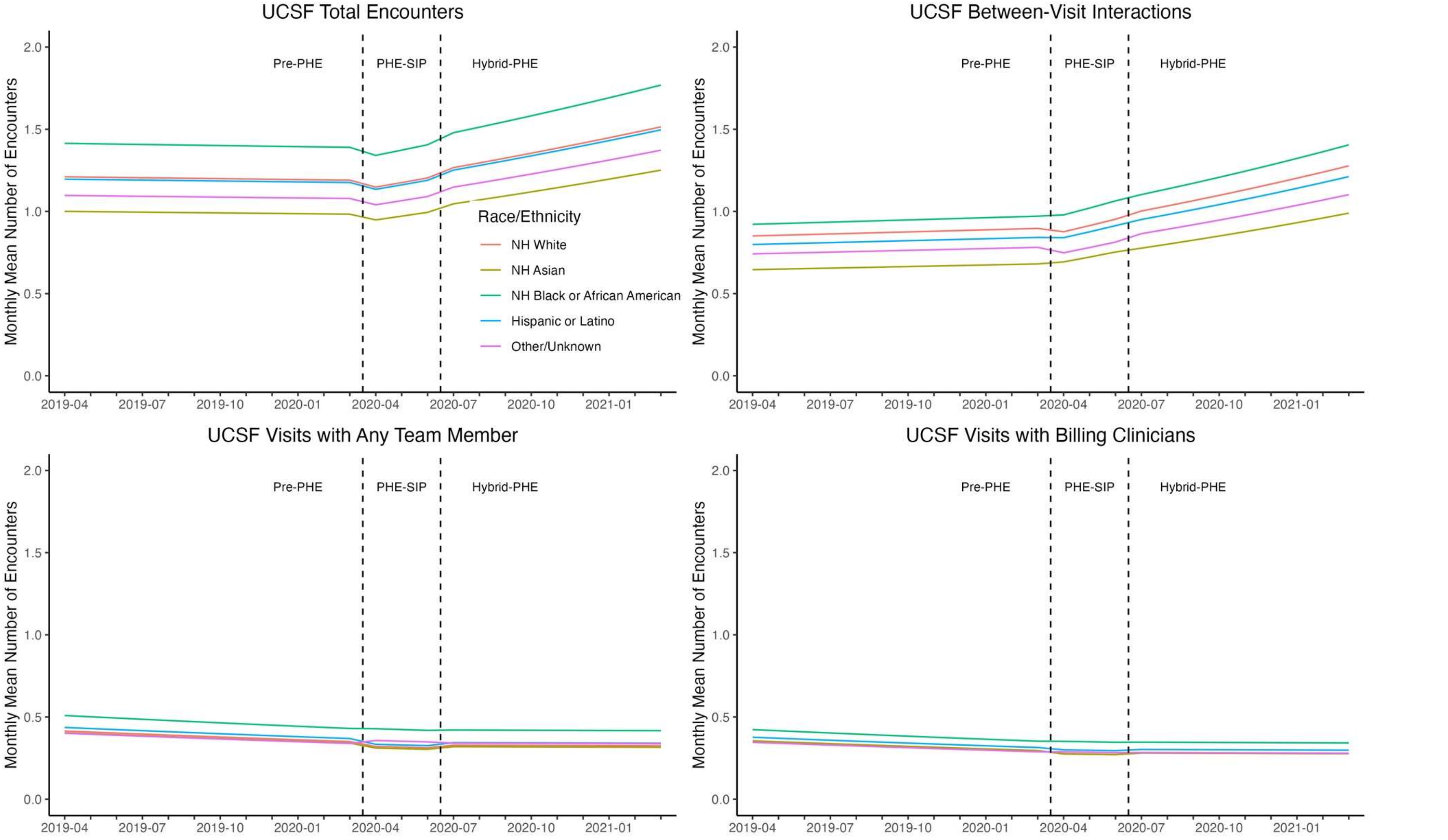

Source Authors' analysis of electronic health record data.

Notes Results are adjusted for sex, age group, language, neighborhood socioeconomic status, health insurance type, Charlson co-morbidity index, baseline hemoglobin A1c, baseline blood pressure, and enrollment in the patient portal. Pre-PHE = Pre COVID-19 Public Health Emergency (4/1/2019-3/31/2020). PHE-SIP = Public Health Emergency Shelter in Place (4/1/2020-6/30/2020). Hybrid-PHE = Hybrid Public Health Emergency (7/1/2020-3/31/2021).

© 2023 Nouri S et al. *JAMA Network Open.*

eFigure 5. Interrupted Time-Series Analysis of Patient-Clinician Encounters per Month at SFHN (n = 8975) Stratified by Patient Race and Ethnicity

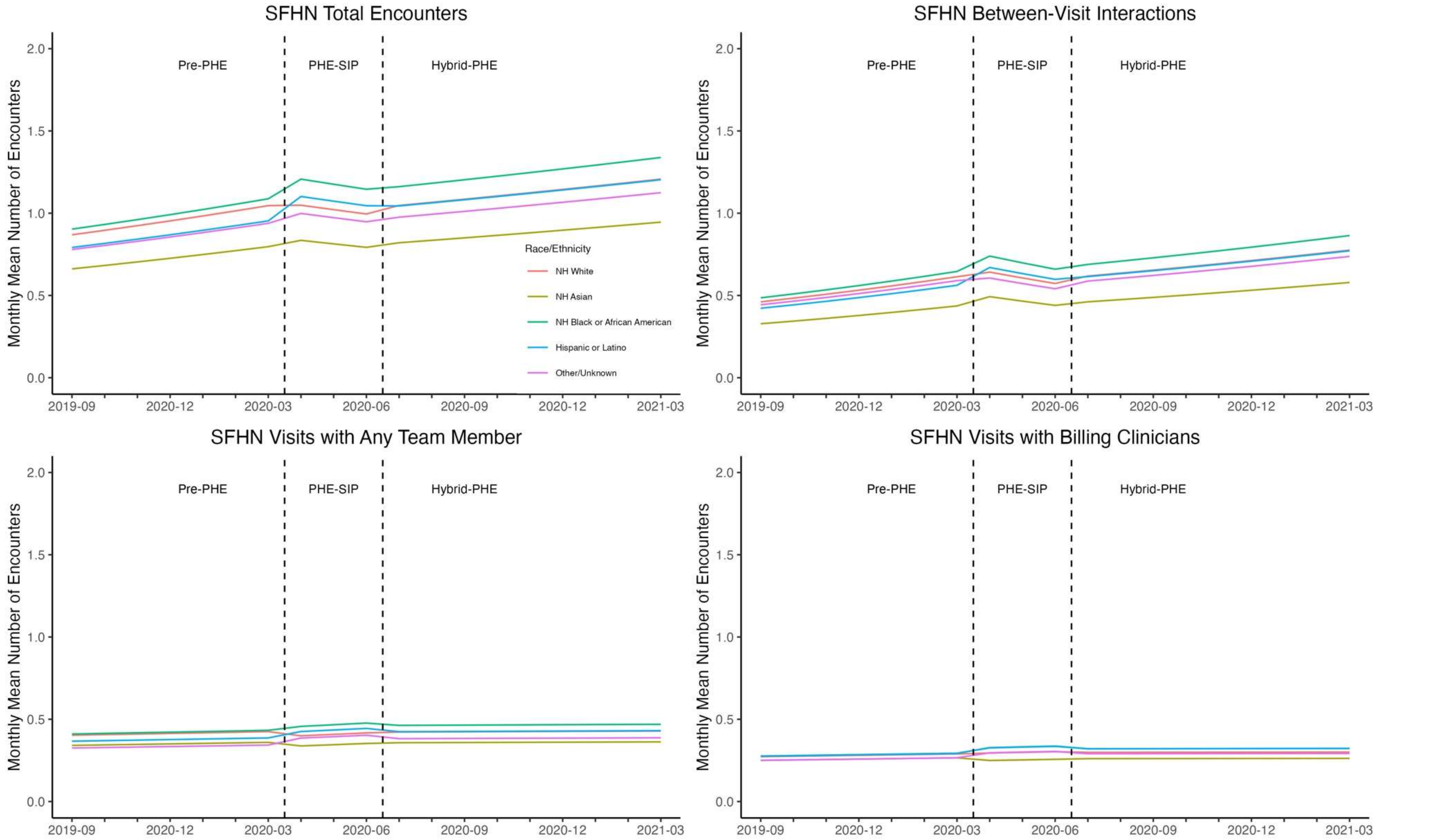

Source Authors' analysis of electronic health record data.

Notes Results are adjusted for sex, age group, language, neighborhood socioeconomic status, health insurance type, Charlson co-morbidity index, baseline hemoglobin A1c, baseline blood pressure, and enrollment in the patient portal. Pre-PHE = Pre COVID-19 Public Health Emergency (4/1/2019-3/31/2020). PHE-SIP = Public Health Emergency Shelter in Place (4/1/2020-6/30/2020). Hybrid-PHE = Hybrid Public Health Emergency (7/1/2020-3/31/2021).

© 2023 Nouri S et al. *JAMA Network Open.*

eFigure 6. Interrupted Time-Series Analysis of Patient-Clinician Encounters per Month at UCSF (n = 4967) Stratified by Patient Language

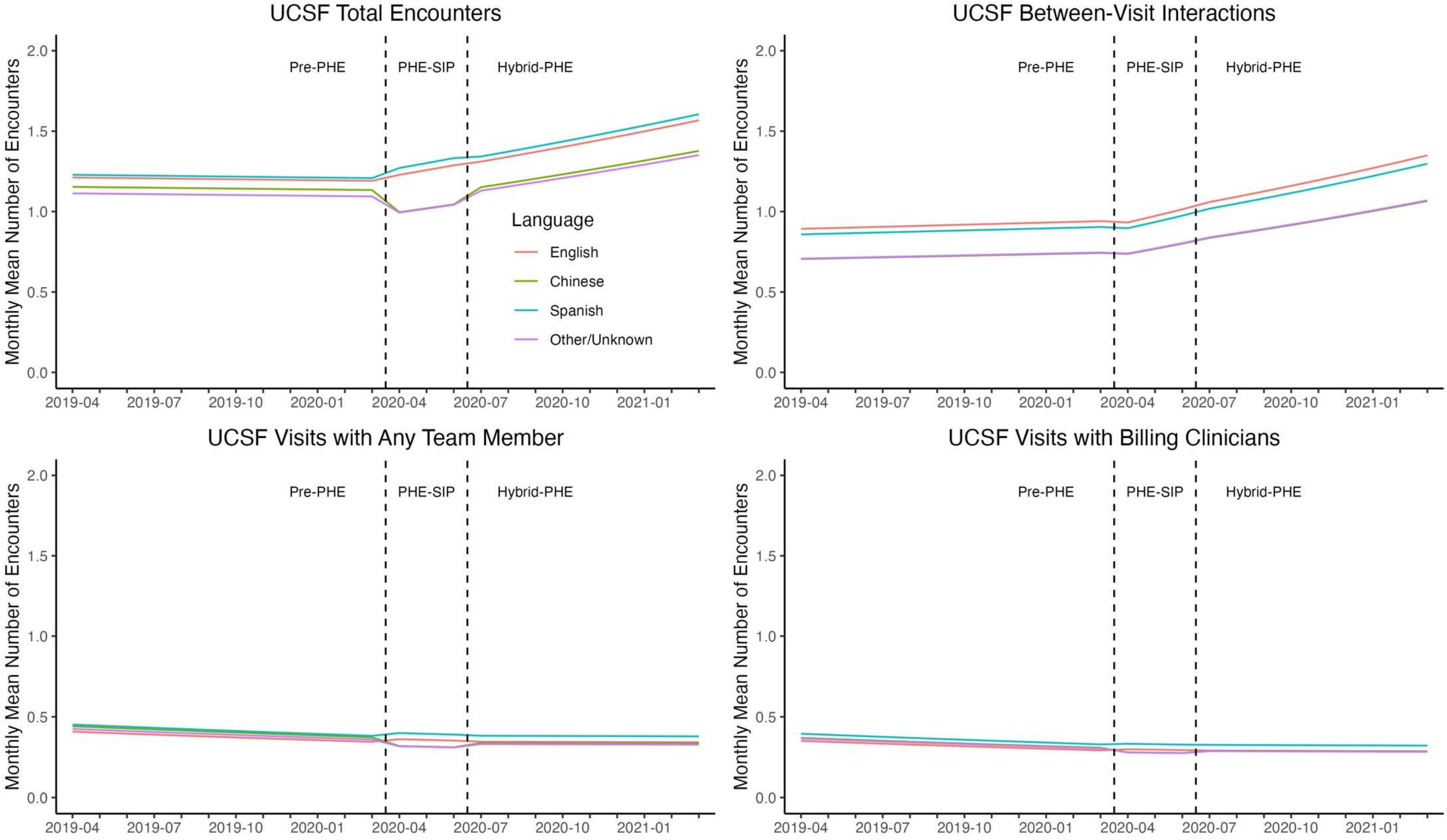

Source Authors' analysis of electronic health record data.

Notes Results are adjusted for age, sex, race/ethnicity, neighborhood socioeconomic status, health insurance type, Charlson co-morbidity index, baseline hemoglobin A1c, baseline blood pressure, and enrollment in the patient portal. Pre-PHE = Pre COVID-19 Public Health Emergency (4/1/2019-3/31/2020). PHE-SIP = Public Health Emergency Shelter in Place (4/1/2020-6/30/2020). Hybrid-PHE = Hybrid Public Health Emergency (7/1/2020-3/31/2021).

© 2023 Nouri S et al. *JAMA Network Open.*

eFigure 7. Interrupted Time-Series Analysis of Patient-Clinician Encounters per Month at SFHN (n = 8975) Stratified by Patient Language

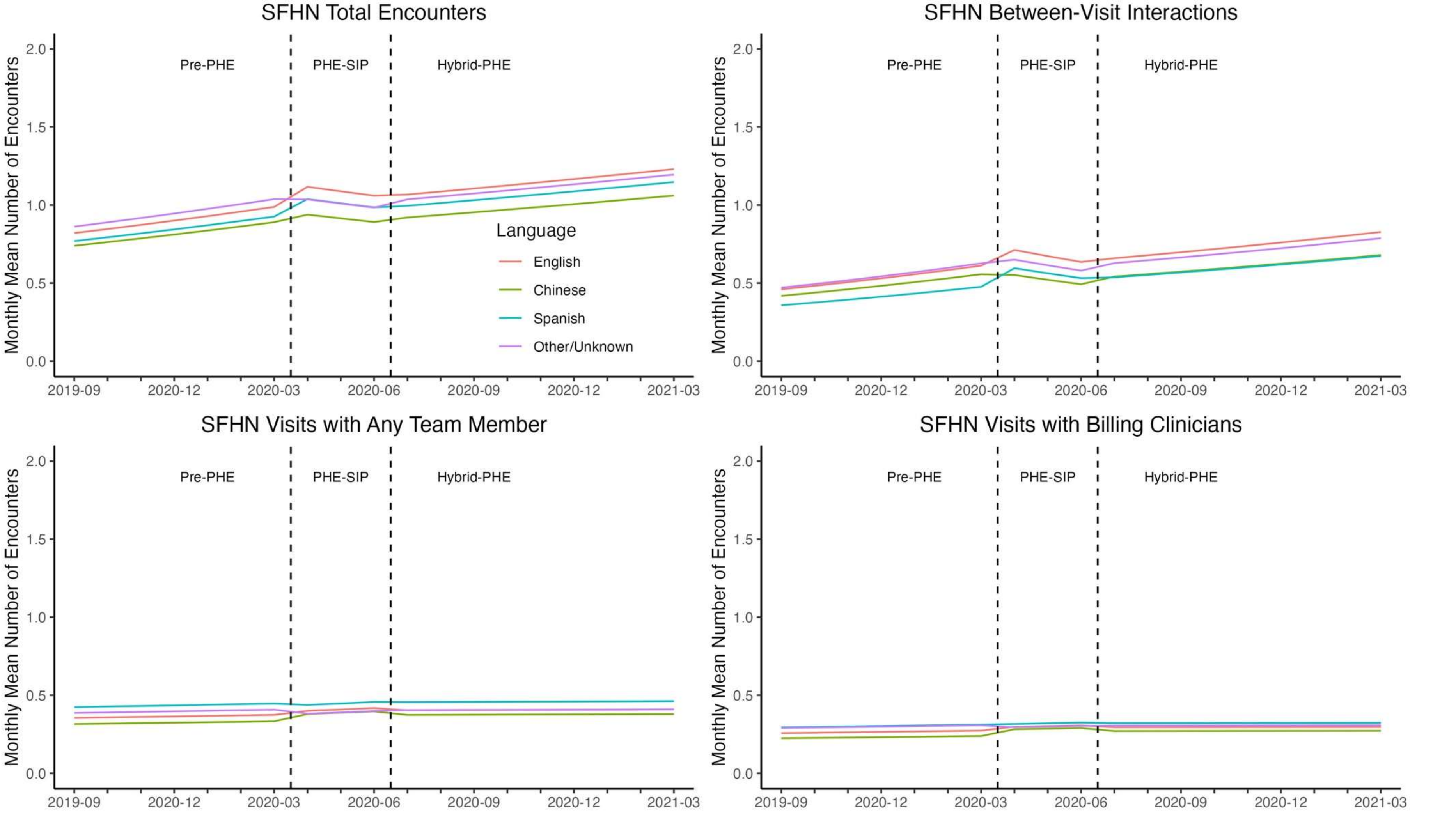

Source Authors' analysis of electronic health record data.

Notes Results are adjusted for age, sex, race/ethnicity, neighborhood socioeconomic status, health insurance type, Charlson co-morbidity index, baseline hemoglobin A1c, baseline blood pressure, and enrollment in the patient portal. Pre-PHE = Pre COVID-19 Public Health Emergency (4/1/2019-3/31/2020). PHE-SIP = Public Health Emergency Shelter in Place (4/1/2020-6/30/2020). Hybrid-PHE = Hybrid Public Health Emergency (7/1/2020-3/31/2021).

© 2023 Nouri S et al. *JAMA Network Open.*

eFigure 8. Adjusted Encounter Means by Age in Pre-PHE and Hybrid-PHE Periods at UCSF (n = 4967) and SFHN (n = 8975)

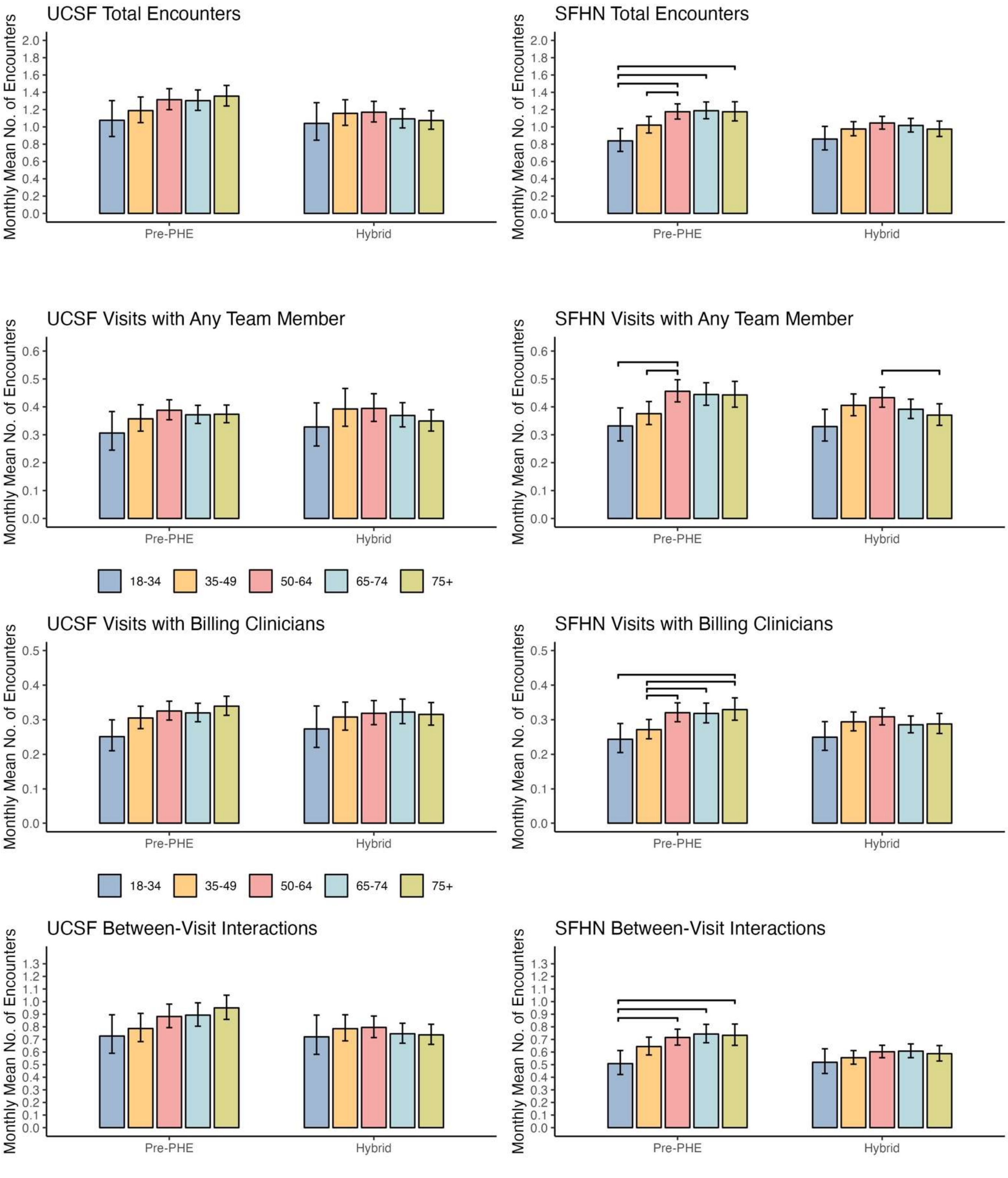

Caption: Pre-PHE = Pre COVID-19 Public Health Emergency (4/1/2019-3/31/2020 at UCSF; 9/1/2019-3/31/2020 at SFHN). Hybrid-PHE = Hybrid Public Health Emergency (7/1/2020-3/31/2021). Bars indicate significant difference at $p < 0.05$.

© 2023 Nouri S et al. *JAMA Network Open.*

eFigure 9. Adjusted Encounter Means by Race and Ethnicity for Visits With Any Team Member in Pre-PHE and Hybrid-PHE Periods at UCSF (n = 4967) and SFHN (n = 8975)

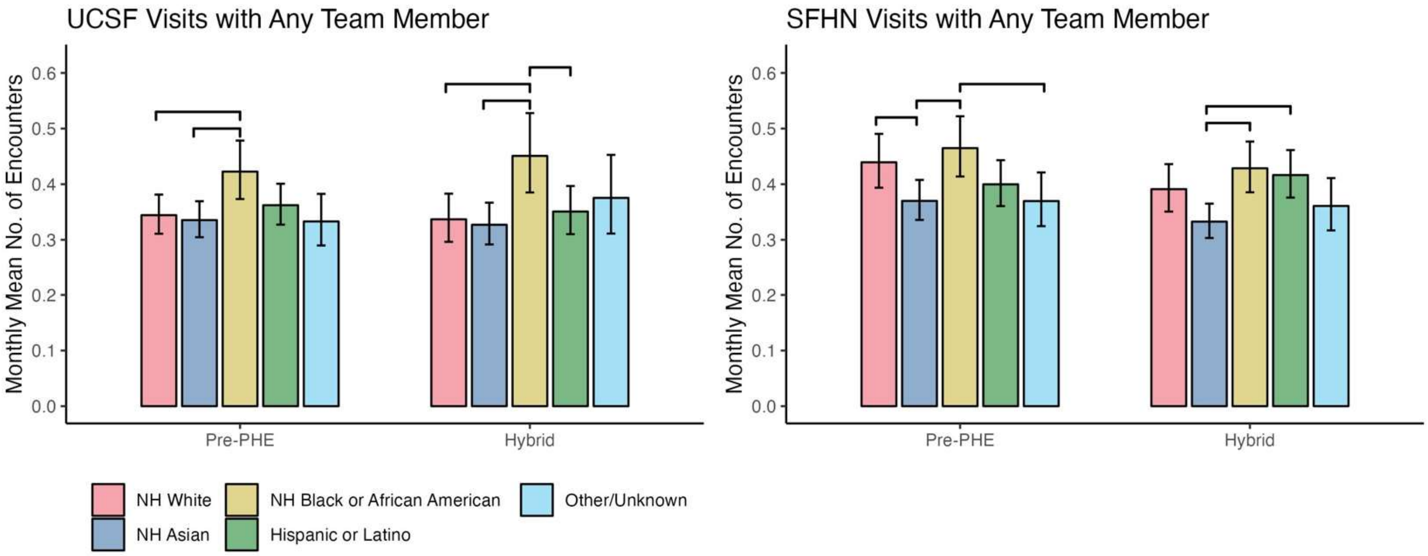

Caption: Pre-PHE = Pre COVID-19 Public Health Emergency (4/1/2019-3/31/2020 at UCSF; 9/1/2019-3/31/2020 at SFHN). Hybrid-PHE = Hybrid Public Health Emergency (7/1/2020-3/31/2021). Bars indicate significant difference at $p < 0.05$.

© 2023 Nouri S et al. *JAMA Network Open.*

eFigure 10. Adjusted Encounter Means by Language for Visits With Any Team Member in Pre-PHE and Hybrid-PHE Periods at UCSF (n = 4967) and SFHN (n = 8975)

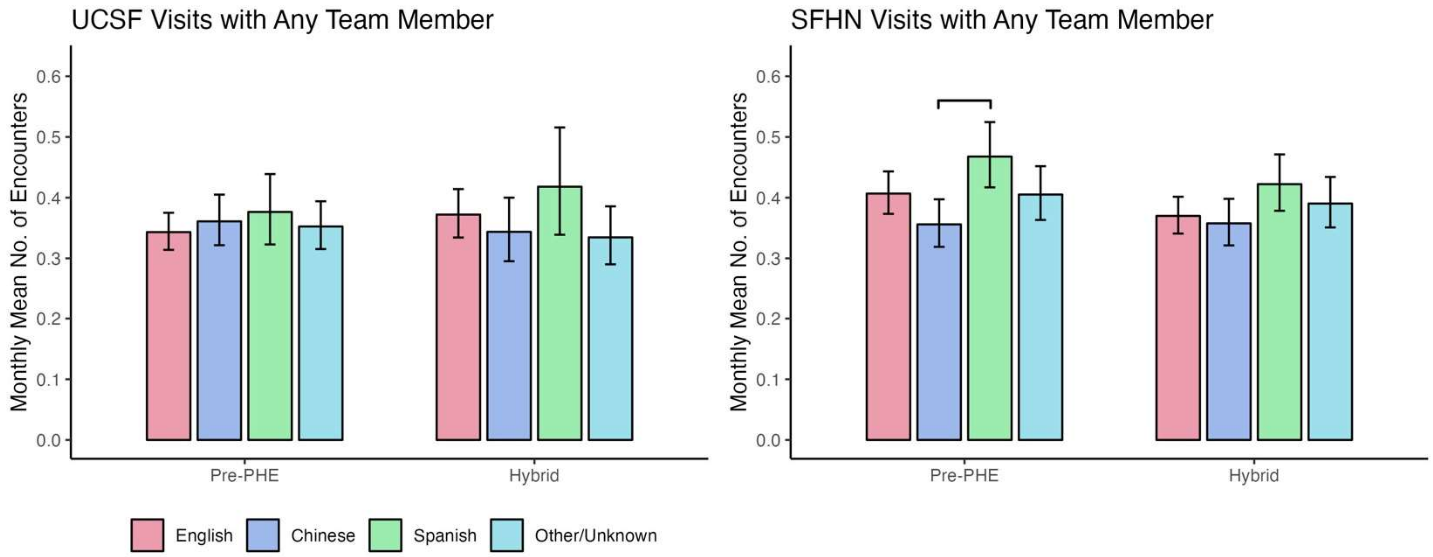

Caption: Pre-PHE = Pre COVID-19 Public Health Emergency (4/1/2019-3/31/2020 at UCSF; 9/1/2019-3/31/2020 at SFHN). Hybrid-PHE = Hybrid Public Health Emergency (7/1/2020-3/31/2021). Bars indicate significant difference at $p < 0.05$.

© 2023 Nouri S et al. *JAMA Network Open.*
