# Supplementary material for: Visit and Between-Visit Interaction Frequency Before and After COVID-19 Telehealth Implementation
Source: JAMA Netw Open. 2023 Sep 15;6(9):e2333944. doi: 10.1001/jamanetworkopen.2023.33944 (PMC10504619; doi:10.1001/jamanetworkopen.2023.33944)
Supplement: Supplement 2. — Data Sharing Statement [file jamanetwopen-e2333944-s002.pdf]

Data Sharing Statement

Nouri. Visit and Between-Visit Interaction Frequency Before and After COVID-19 Telehealth Implementation. *JAMA Netw Open*. Published September 15, 2023. doi:10.1001/jamanetworkopen.2023.33944

Data
Data available: No

Additional Information
Explanation for why data not available: Data will be made available upon reasonable request to corresponding author. We did not specify that we would be sharing de-identified data publicly in our IRB.
